# New haplochromine cichlid from the upper Miocene (9–10 MYA) of Central Kenya

**DOI:** 10.1186/s12862-020-01602-x

**Published:** 2020-06-05

**Authors:** Melanie Altner, Bernhard Ruthensteiner, Bettina Reichenbacher

**Affiliations:** 1 Department of Earth and Environmental Sciences, Paleontology and Geobiology, Ludwig-Maximilians-Universität München, Richard-Wagner-Strasse 10, 80333 Munich, DE Germany; 2 Section Evertebrata Varia, SNSB – ZSM Bavarian State Collection of Zoology, Münchhausen-Strasse 21, 81247 Munich, DE Germany; 3 GeoBio-Center at Ludwig-Maximilians-Universität München, Richard-Wagner-Strasse 10, 80333 Munich, DE Germany

## Abstract

**Background:**

The diversification process known as the Lake Tanganyika Radiation has given rise to the most speciose clade of African cichlids. Almost all cichlid species found in the lakes Tanganyika, Malawi and Victoria, comprising a total of 12–16 tribes, belong to this clade. Strikingly, all the species in the latter two lakes are members of the tribe Haplochromini, whose origin remains unclear. The ‘out of Tanganyika’ hypothesis argues that the Haplochromini emerged simultaneously with other cichlid tribes and lineages in Lake Tanganyika, presumably about 5–6 million years ago (MYA), and that their presence in the lakes Malawi and Victoria and elsewhere in Africa today is due to later migrations. In contrast, the ‘melting pot Tanganyika hypothesis’ postulates that Haplochromini emerged in Africa prior to the formation of Lake Tanganyika, and that their divergence could have begun about 17 MYA. Haplochromine fossils could potentially resolve this debate, but such fossils are extremely rare.

**Results:**

Here we present a new fossil haplochromine from the upper Miocene site Waril (9–10 million years) in Central Kenya. Comparative morphology, supported by Micro-CT imaging, reveals that it bears a unique combination of characters relating to dentition, cranial bones, caudal skeleton and meristic traits. Its most prominent feature is the presence of exclusively unicuspid teeth, with canines in the outer tooth row. †*Warilochromis unicuspidatus* gen. et sp. nov. shares this combination of characters solely with members of the Haplochromini and its lacrimal morphology indicates a possible relation to the riverine genus *Pseudocrenilabrus*. Due to its fang-like dentition and non-fusiform body, †*W. unicuspidatus* gen. et sp. nov. might have employed either a sit-and-pursue or sit-and-wait hunting strategy, which has not been reported for any other fossil haplochromine cichlid.

**Conclusions:**

The age of the fossil (9–10 MYA) is incompatible with the ‘out of Tanganyika’ hypothesis, which postulates that the divergence of the Haplochromini began only 5–6 MYA. The presence of this fossil in an upper Miocene palaeolake in the Central Kenya Rift, as well as its predatory lifestyle, indicate that Haplochromini were already an important component of freshwater drainages in East Africa at that time.

## Background

**Fig. 1:**
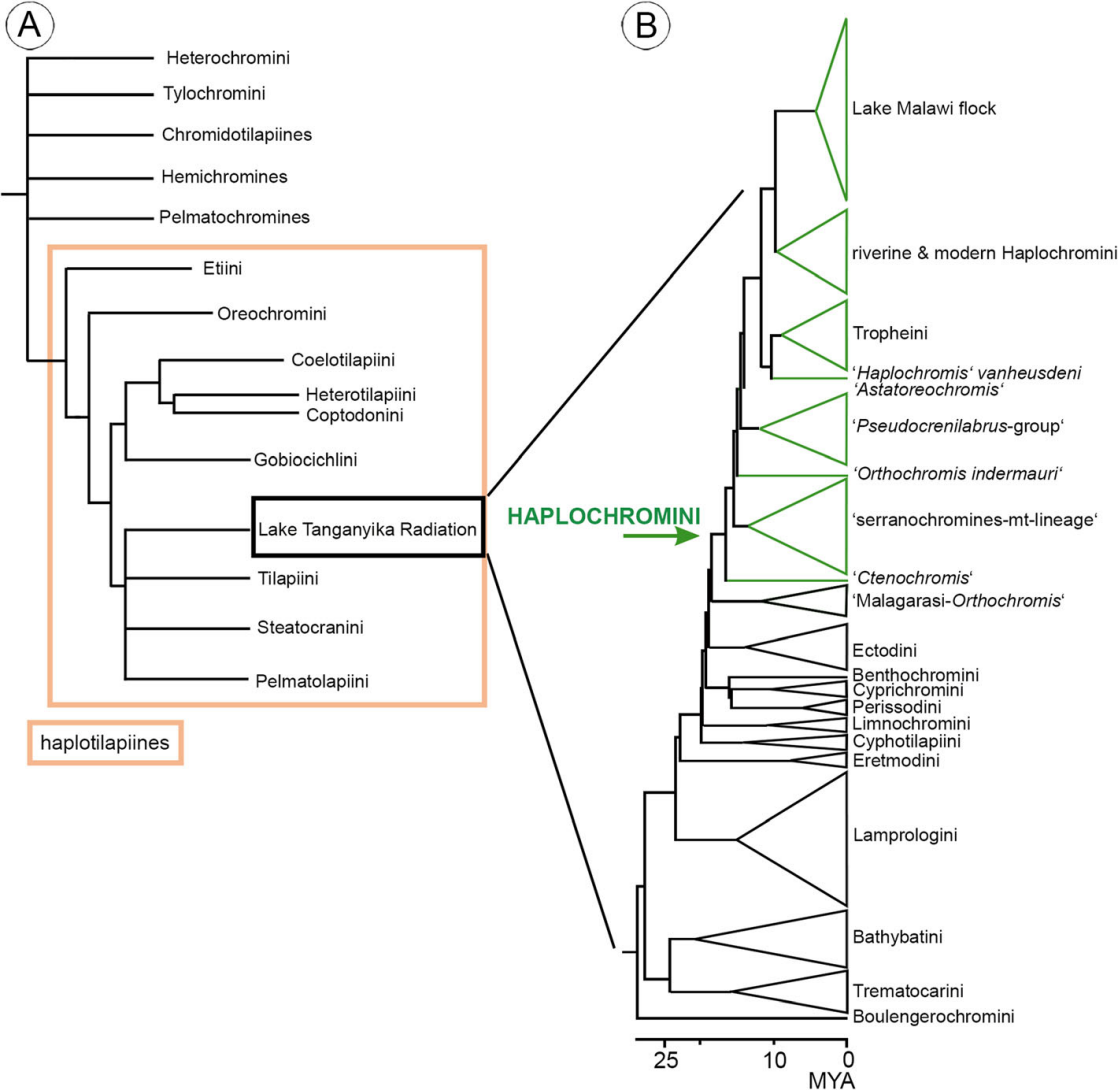
**a** Simplified composite phylogenetic tree depicting possible relationships among the Pseudocrenilabrinae, based on Schwarzer et al. [14] and Dunz and Schliewen [15] (reused with slight modifications from Altner et al., [16] (open access article distributed under CC-BY-NC-ND 4.0 license; https://creativecommons.org/licenses/by-nc-nd/4.0/)); **b** Time calibrated phylogeny of all lineages comprising the Lake Tanganyika Radiation (re-drawn after Schedel et al., [17] distributed under CC-BY license (https://creativecommons.org/licenses/by/4.0/), simplified, error bars for ages of nodes not shown). The area of each triangle corresponds to the number of species used in the original publication; all lineages of the Haplochromini are depicted in green

Cichlidae are one of the most species-rich freshwater fish families, with about 1700 valid species having been recognized to date [1, 2], but their estimated species number may be as high as 3000–4000 [3]. They have been intensively studied and are especially famous for their capacity for rapid adaptive speciation (e.g., [4–8]). The most remarkable example of this ability is found in the Great Lakes of the East African Rift System, i.e. Lake Tanganyika, Lake Malawi and Lake Victoria, and is referred to as the ‘Lake Tanganyika Radiation’ or ‘East African Radiation’ (e.g., [3, 9–11]). Depending on the author consulted, the Lake Tanganyika Radiation comprises 12 to 16 tribes or lineages ([12, 13]; Fig. 1), most of them are endemic to the Great Lakes. Exceptions are members of the tribe Lamprologini, which are also represented in rivers across East and Central Africa [18–20], and species of the tribe Haplochromini, which are distributed in rivers and lakes all over Africa, but reach their highest levels of diversity in Lakes Victoria and Malawi (e.g., [18, 21–24]).

With about 1700 species, the Haplochromini is the most speciose of the groups that contributed to the Lake Tanganyika Radiation (see [25]). The tribe can be subdivided into several lineages, of which the flock found in Lake Victoria and the neighbouring lakes (Edward, George and Kivu) is considered to be a superflock [26] (Fig. 1b; see also [17] and [27]). In addition, all Haplochromini are maternal mouthbrooders and have evolved numerous specialized adaptations and feeding strategies (e.g., [28–30]). The Haplochromini that are endemic to Lake Tanganyika, i.e. the Tropheini, are either herbivores (e.g., [31]) or insectivores [32–34], but the haplochromine species of Lake Malawi and Victoria display the full range of feeding specializations from ‘Aufwuchs’ feeding (grazing on algal communities that are attached to rocks) through insectivory, plankton-feeding, piscivory, herbivory, mollusc-feeding and death feigning to lepidophagy and paedophagy (e.g., [35–40]).

Even though the Haplochromini have been the subject of a very large number of studies dealing with their ecology, behavior or trophic specializations (e.g., [41–44]), many issues remain to be resolved. One of the central questions concerns the evolutionary history of the Haplochromini. Two contrasting hypotheses have been proposed. One theory postulates that the Haplochromini originated within Lake Tanganyika, presumably about 5–6 MYA [25, 45]. The other suggests that the emergence of the tribe predates the formation of Lake Tanganyika (the ‘melting pot Tanganyika’ hypothesis of Weiss et al. [46]) and that their divergence age could be as old as 17 MYA (see [17] and Fig. 1b). This second hypothesis is compatible with the proposal that at least four different riverine lineages of Haplochromini (Tropheini, *Pseudocrenilabrus*, *Astatoreochromis*, and *Astatotilapia*) have independently colonized Lake Tanganyika (see [11]). In addition, the phylogenetic reconstruction by Schedel et al. [17] shows that the closest extant relatives of the Haplochromini all live in habitats that lie to the east of Lake Tanganyika: Four species of the paraphyletic genus *Orthochromis*, which are sister to the Haplochromini, thrive in the Malagarasi river system, while *Ctenochromis pectoralis*, which is sister to the remaining Haplochromini, is endemic to drainage systems in Kenya and Tanzania (see Fig. 1b). Thus, the authors suggest that the most recent common ancestor of the Haplochromini must have lived east of Lake Tanganyika.

Fossil cichlids have the potential to clarify the evolutionary history of the group, because they can provide solid age constraints for a given lineage or tribe, and their biogeographic distribution can provide support for one or other of the competing hypotheses. However, the assignment of a fossil cichlid at the level of tribe has proven to be very difficult, because features of the skeleton may show little variation between tribes. The objective of this study is to present a newly discovered cichlid fossil from the upper Miocene Ngorora Formation (Central Kenya) and to infer some aspects of its feeding strategy.

### Geological setting

The Tugen Hills are part of the eastern branch of the East African Rift System (see e.g., [47–50]). The mountain range extends for about 100 km from north to south [51, 52] and its maximum altitude is around 2400 m. Its thick (up to 3000 m) successions of volcanic, fluvial and lacustrine rocks document active volcanism and the development of deep lakes as the result of ongoing rifting activity [53–55]. Today, the rock deposits exposed in the Tugen Hills represent the most complete fossiliferous record of the Miocene-Pliocene Epoch in Africa [52] and have been the focus of many research projects, e.g. dealing with regional climate change (e.g., [49, 56, 57]), vegetation (e.g., [58–62]) and the evolution of mammals and hominids (e.g., [63–67]). References to its fossil fish record are generally restricted to comparatively brief remarks in older publications (e.g., [53, 54, 68–73]). However, this topic has received renewed attention in recent years [16, 55, 74–76]. Most of the newly described fish fossils have been discovered in the ‘fossil fish Lagerstätte’ of the middle-to-upper Miocene Ngorora Formation (13.3–9 MYA) (see [55]).

### Study site

**Fig. 2:**
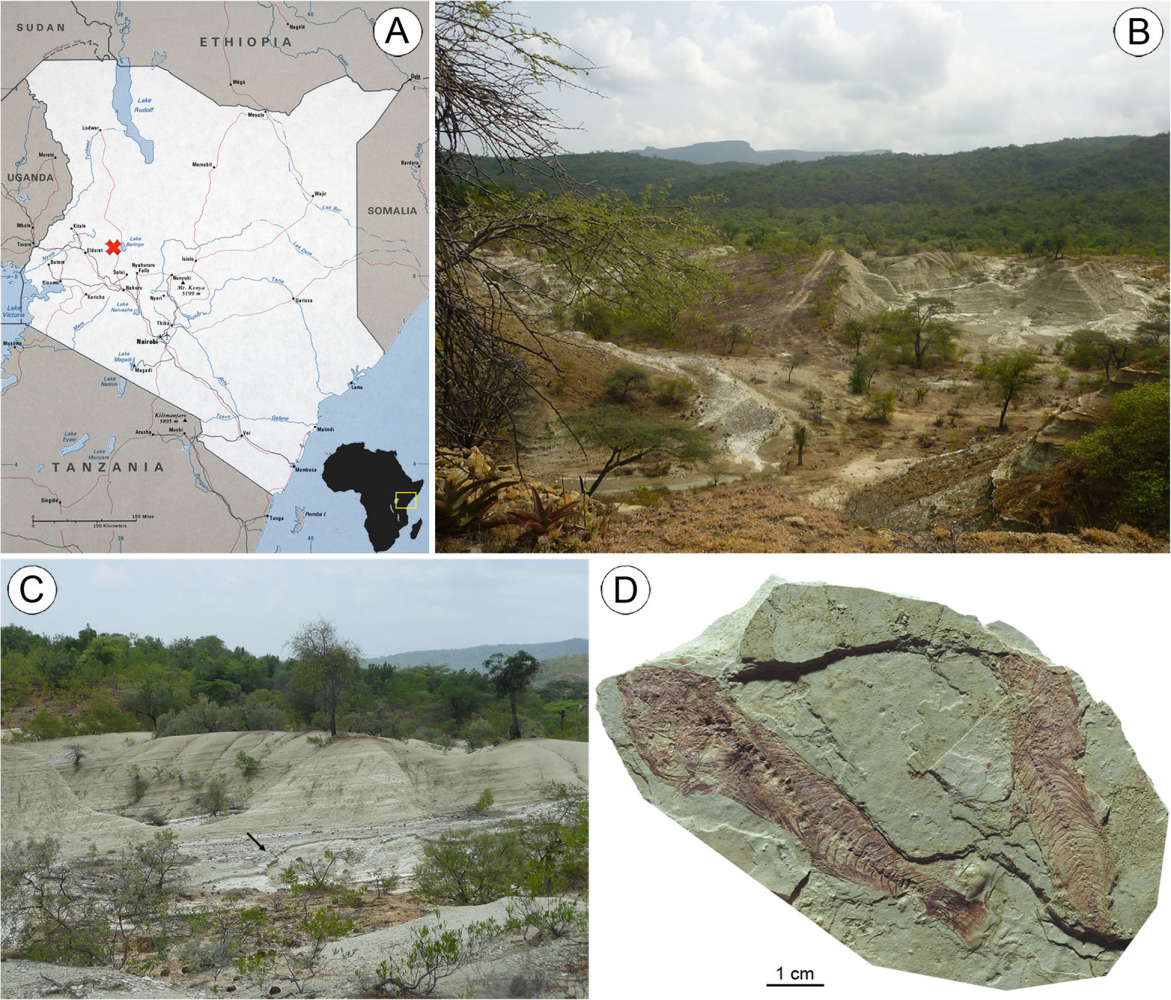
**a** Location of the fossiliferous beds at the Waril site (red cross) in Kenya (source of map: copyright 2019 Mapsland; mapsland.com with terms of Creative Commons Attribution-shareAlike 3.0 license [CC BY-SA 3.0] https://creativecommons.org/licenses/by-sa/3.0/); **b-c** Upper Miocene lacustrine sediments exposed at Waril (arrow in **c** points to fish-bearing layer); **d** Example of sediment block containing fish fossils (OCO-5-13). Photos **b** and **c** were taken by first author, photo **d** by M. Schellenberger (SNSB-BSPG, Bavarian State Collection Palaeontology and Geology, Munich, Germany). Copyright (2020), with permission from SNSB – BSPG

The fossil specimen described here derives from the Ngorora Formation (Fm) at the site Waril (0°40′56.21″N; 35°43′7.43″E). This site is located in a remote area 4 km south of Barwesa and 8 km northeast of Kapturwo in Baringo County, Kenya (Fig. 2a). The name ‘Waril’ derives from a Tugen term meaning ‘at the white place’ [77] and probably refers to the light colour of the sediments (Fig. 2b–d). The exposed sediments comprise tuffs and claystones and represent a late Miocene palaeolake (9–10 MYA, see [55]). That numerous very well-preserved cichlid fish fossils occur at Waril has been known for a long time [72], but the locality has only recently become the subject of detailed investigations, because new excavations could be undertaken in 2013 and 2014. Among the material recovered, two particular fossil specimens were found to be unique. †*Tugenchromis pickfordi* Altner, Schliewen, Penk & Reichenbacher, 2017 has already been described as a stem-group member of the Lake Tanganyika Radiation [16], and the other most striking specimen is presented in this study. The rest of the material is currently under study.

## Results

The fossil presents features which, in combination, are typical for modern cichlid fishes (see [78–84]): an interrupted lateral line; caudal fin skeleton with eight principal fin rays in each lobe, two epural bones, uroneural probably autogenous, autogenous parhypural, preural centrum 2 with autogenous haemal spine and reduced neural spine, haemal spine of preural centrum 3 not autogenous; presence of five branchiostegals; single dorsal fin consisting of spines and rays; pelvic fin with one spine and five rays (for details see below).

There is no unambiguous synapomorphy known for the subfamily Pseudocrenilabrinae. The putative synapomorphy ‘strongly pigmented opercular spot’, proposed by Stiassny [85], is not present in *Heterochromis* – and would not be recognizable in a fossil in any case. A ‘simple sutural union between the vomerine wing and the parasphenoid’ was identified as typical for the Pseudocrenilabrinae by Stiassny [85], but she already noted that exceptions exist (e.g. *Heterochromis*). In addition, two members of the subfamily Ptychochrominae, i.e. *Ptychochromis* and *Paratilapia*, and some members of the subfamily Etroplinae possess this character [85]. On inspection of the morphological data matrix compiled by Stiassny [85], the character ‘single supraneural’ appears to be a putative synapomorphy for the Pseudocrenilabrinae, but this character also occurs in the Neotropical cichlids (Cichlinae) (see e.g. Kullander, [86]).

**Fig. 3:**
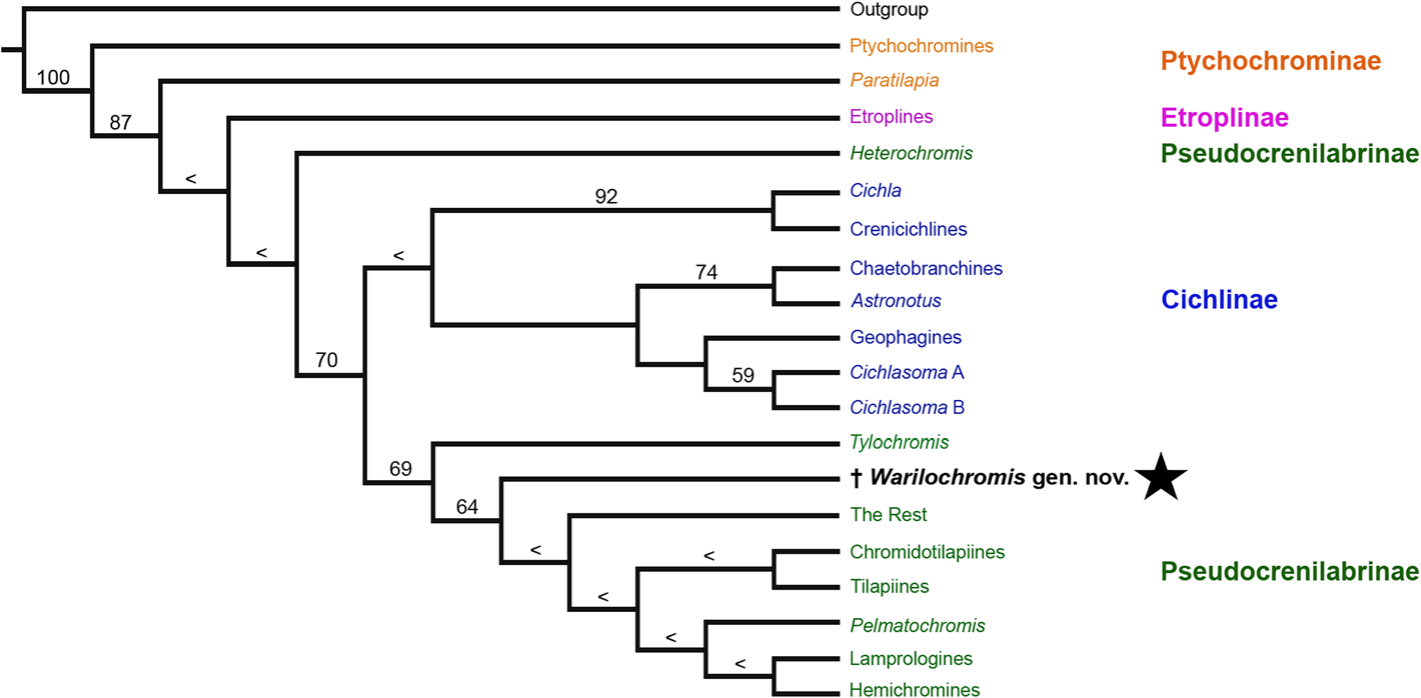
Phylogenetic position of †*Warilochromis unicuspidatus* gen. et sp. nov. (highlighted in bold) among the four cichlid subfamilies based on the slightly modified morphological data matrix of Stiassny [85] (see Methods for details). This is the single most parsimonious tree produced by TNT (implied weights, K = 12), tree length = 33 steps, consistency index = 0.85, retention index = 0.93. Bootstrap values from 1000 pseudoreplicates are presented on the branches. The arrowhead symbols (<) indicate values below 50%. Four (out of 28) characters were coded for †*Warilochromis* gen. nov

To tentatively assign the new fossil to one of the subfamilies of the Cichlidae we carried out a maximum-parsimony analysis of the matrix based on Stiassny [85] using implied weighting (K = 12.0). The resulting single most parsimonious tree (MPT) is shown in Fig. 3. This tree shows a higher resolution than the original phylogeny of Stiassny [85], which is probably due to the use of implied weighting. If the analysis is run with all characters set unweight, three MPTs are obtained and the resulting consensus tree matches exactly the original tree by Stiassny [85]. Stiassny’s Ptychochromines emerges as sister to all Cichlidae, the Etroplinae (Stiassny’s Etroplines) are sister to all Cichlidae except the Ptychochrominae (Stiassny’s Ptychochromines + *Paratilapia*), and *Heterochromis* is sister to all Cichlidae except the Ptychochrominae and Etroplinae (=Madagascan and Indian taxa). The relationships within the Cichlinae (Neotropical cichlids) are resolved with moderate support (this clade was polyphyletic in the original tree obtained by Stiassny, [85]). Also, the clade comprising the Pseudocrenilabrinae except *Heterochromis* is resolved, albeit with low support. The new fossil specimen is placed within the latter clade (including the African cichlids *Tylochromis*, Hemichromines, Chromidotilapiines, *Pelmatochromis*, Lamprologines and ‘The Rest’) with low support and is sister to all African cichlids except *Tylochromis* and *Heterochromis*.

SYSTEMATIC PALAEONTOLOGY.

SERIES OVALENTARIA Wainwright et al., 2012.

SUPERORDER CICHLOMORPHAE Betancur-R. et al., 2013.

ORDER CICHLIFORMES Nelson et al., 2016.

FAMILY CICHLIDAE Bonaparte, 1835.

SUBFAMILY PSEUDOCRENILABRINAE Fowler, 1934.

GENUS †*WARILOCHROMIS* gen. nov.

**Zoobank Nr**.: LSID urn:lsid:zoobank.org:act:FD3D44E6–A313–4809–8FDF–46850A20B5E1.

**Generic Diagnosis**—†*Warilochromis* differs from all other fossil and extant cichlids in a unique combination of characters comprising the following: four lateral-line tubules on the lacrimal bone; ascending process of premaxilla shorter than horizontal ramus; oral dentition unicuspid with large canines in the outer row and smaller teeth in the inner row; one supraneural bone; 33 (19 + 14) vertebrae; vertebra 17 associated with pterygiophore of last dorsal fin spine; three anal fin spines; hypural 1 + 2 fused and autogenous, hypural 3 + 4 fused and probably fused to terminal centrum; divided lateral line; cycloid scales.

**Etymology**—Name refers to the locality Waril where the fossil was found. The Greek word ‘Chromis’ (χρόμις) is a name used by the Ancient Greeks and has been applied to various fish. It is a common second element in cichlid genus names.

**Type Species**—†*Warilochromis unicuspidatus* sp. nov.

†*WARILOCHROMIS UNICUSPIDATUS* sp. nov.

**Holotype**—2014-WA-16. Skeleton preserved in left lateral view; total length 8.2 cm, standard length 6.9 cm, and body length approximately 4.6 cm. Bones of skeleton almost completely preserved, with exception of the first four abdominal vertebrae, caudal vertebrae 4–6, and preural centrum 2 of which only imprints are visible. For taphonomic reasons, the long axis of the specimen is shortened.

**Diagnosis**—Same as for the genus.

**Etymology**—The specific name ‘*unicuspidatus*’ refers to the latin words ‘*unus*’ = one and ‘*cuspis*’ = point, to emphasize the conspicuous dentition of the oral jaws.

**Type locality and age**—Kenya, Tugen Hills, Ngorora basin, Ngorora Formation, Member E, site Waril (0°40′56.21″N; 35°43′7.43″E), ca. 9–10 Ma.

### Description

#### General description

**Fig. 4:**
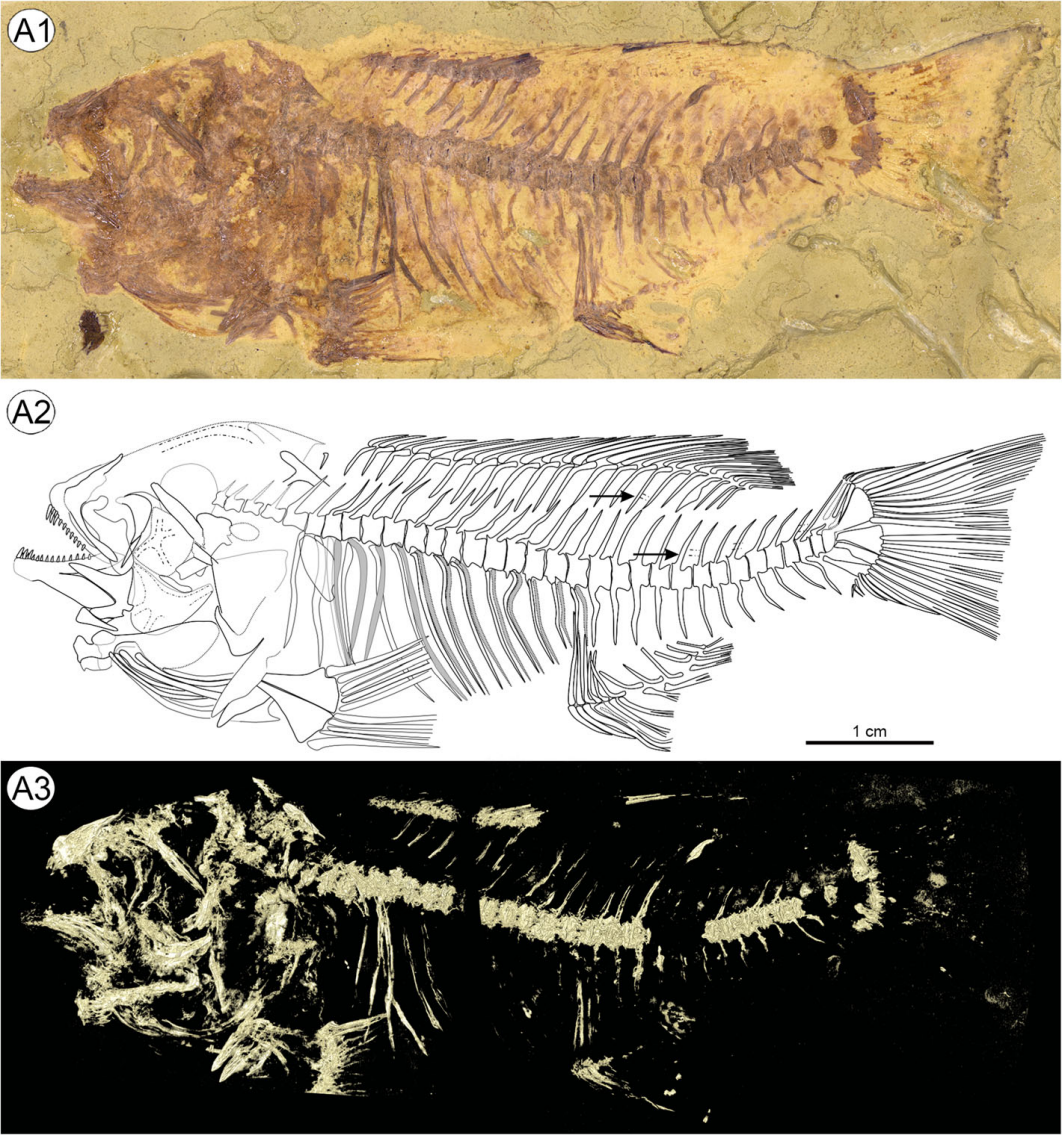
Holotype and single specimen of †*Warilochromis unicuspidatus* gen. et sp. nov. **a1**, Photograph of specimen; **a2**, Interpretative drawing (arrows refer to lateral-line canals of the anterior and posterior lateral-line segments); **a3**, Micro-CT rendering revealing the side of the fossil that was embedded in the sediment (mirrored for ease of comparison). Note that the specimen is distorted and shortened along the anterior-posterior axis for taphonomic reasons. This has led to the displacement of the anteriormost vertebrae and distortion of the shape of the orbit. Photographs by first author

Approximately 82 mm in total length and 69 mm in standard length (SL) (see Table 1). Greatest body depth behind head. Stout body with relatively short but narrow caudal peduncle. Body approximately straight although posterior part of vertebral column is bent upwards slightly (Fig. 4). Large skull (head length 33.3% of SL), terminal snout, probably isognathous jaws, oral dentition unicuspid. Divided lateral line.

**Table 1:** Morphometric and meristic data for †*Warilochromis* gen. nov

Measurement	mm / % of SL	Counts	
Total length	81.9/118.8	Dorsal fin	XIV, 10
Standard length	68.9	Anal fin	III, 9
Body length	45.9/66.6	Pelvic fin	I, 5
Head length	22.9/33.3	Caudal fin	4 + i + 7 + 7 + i + 5
Head depth	23.3/33.8	Vertebrae	33 (19 + 14)
Length of dorsal fin base	31.1/45.2	VtPtLDs	17
Length of anal fin base	12.1/17.5		
Length of pelvic fin base	3.5/5.0		
Length of pelvic fin spine	10.0/14.6		
Length of caudal fin	16.0/23.2		
Maximum body depth	21.7/31.4		
Depth of body at anal fin	19.6/28.4		
Minimum body depth	8.6/12.5		
Predorsal distance	27.5/39.9		
Postdorsal distance	23.2/33.7		
Preanal distance	44.3/64.3		
Length of caudal peduncle	13.4/19.5		
Prepelvic distance	23.4/33.9		
Length of lower oral jaw	9.8/14.3		
Length of premaxillary ascending process	5.6/8.2		
Length of premaxilla	7.2/10.5		

Abbreviation: *VtPtLDs* Ordinal number of the vertebra associated with pterygiophore of last dorsal fin spine

#### Neurocranium and infraorbital series

**Fig. 5:**
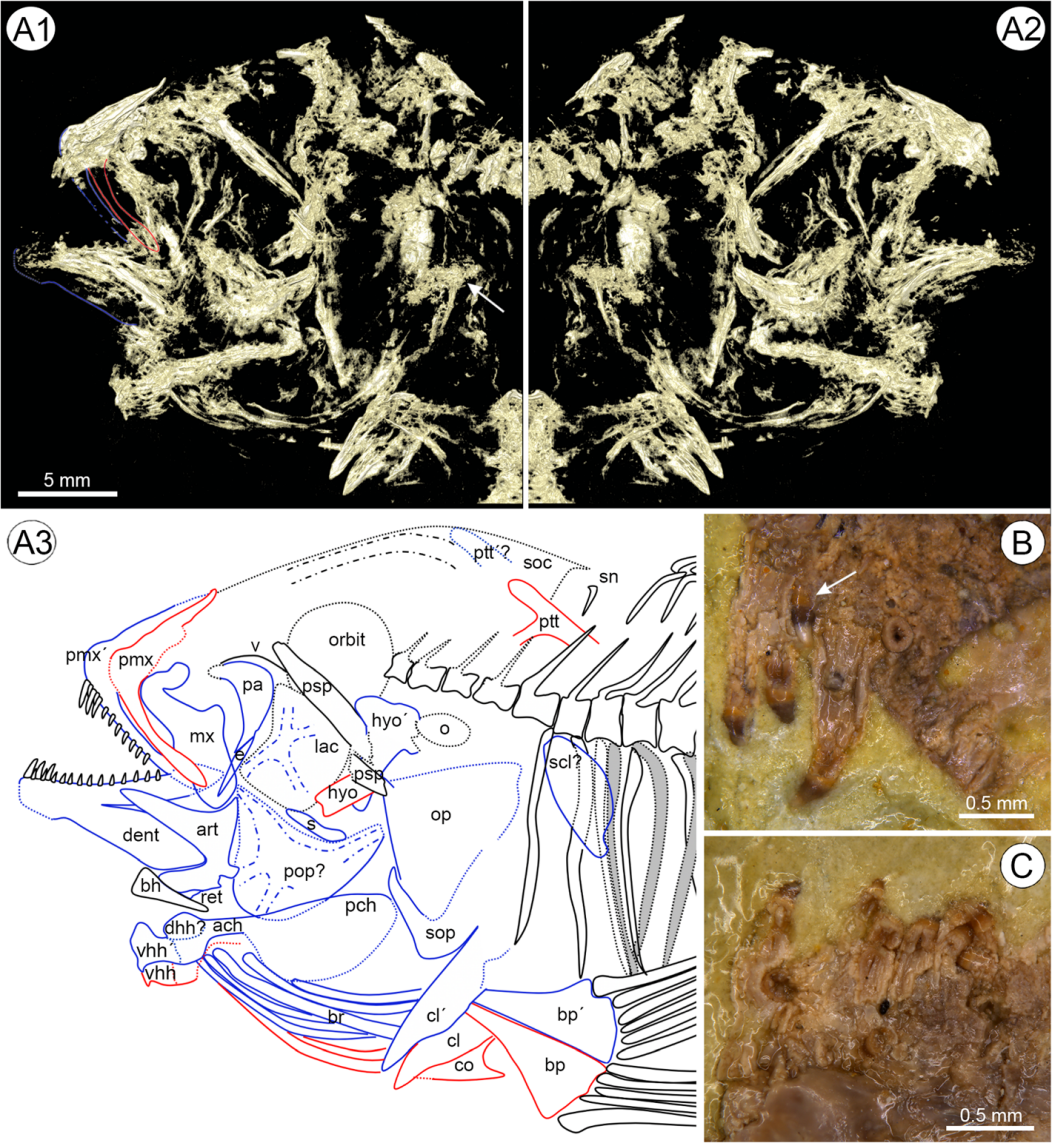
Head and dentition of † *W. unicuspidatus* gen. et sp. nov. **a1–2**, Micro-CT renderings; **a3**, interpretative reconstruction of the head and dentition. The colored lines depict bones that were only recognizable using light microscopy; **b**, Light microscopical close-up of anterior part of right premaxillary (medial view); note that small conical tooth of the inner row (arrow) lies above large caniniform tooth of the outer row; **c**, Light microscopical close-up of anterior part of left dentary with conical teeth. Photographs by first author. Abbreviations: **ach**, anterior ceratohyal; **art**, angulo-articular; **bp & bp´**, basipterygium; **bh**, basihyal; **cl & cl´**, cleithrum; **co**, coracoid; dent, dentary; **dhh**, dorsal hypohyal; **e**, ectopterygoid; **hyo & hyo´**, hyomandibula; **lac & lac´**, lacrimal; **mx**, maxilla; **o**, otolith imprint; **op**, opercle; **pa**, palatine; **pch**, posterior ceratohyal; **pmx & pmx´**, premaxilla; **pop**, preopercle; **psp**, parasphenoid, **ptt & ptt´**, posttemporal; **s**, symplectic; **scl**, supracleithrum; **sn**, supraneural; **sop**, suboperculum; **v**, vomer; **vhh & vhh´**, ventral hypohyal

Outline of neurocranium gently ascending, straight above orbit and slightly convex above supraoccipital crest. Supraoccipital crest low. Frontals unclear, but neurocranial lateral-line canals partially visible (Figs. 4, and Fig. 5a1–3). Massive, straight parasphenoid, broken posteriorly; vomer partially preserved; suture between vomer and parasphenoid simple, not notched (Figs. 5a1–a3).

Infraorbital series comprises the lacrimal (first infraorbital = IO1) (Figs. 5a1–3). Other infraorbital bones are not recognizable, either because the adjacent infraorbital(s) were reduced or because they were lost during fossilization. Lacrimal presents four lateral-line tubules and no scale cover (Figs. 5a1–3).

#### Oral jaws and teeth

Premaxilla slender with ascending process approximately 75% of the length of horizontal ramus (5.6 mm vs. 7.2 mm; see Table 1); left horizontal ramus visible as imprint, right horizontal ramus preserved in medial view with teeth in situ. Recognizable teeth comprise (i) three large canines (length 0.7–1.1 mm) of which the two anteriormost ones are preserved in labial view and do not show lateral compression (Fig. 5b); (ii) a small unicuspid tooth (length 0.4 mm) positioned slightly medial to the largest teeth (indicated by the arrow in Fig. 5b); (iii) a small unicuspid tooth (length 0.2 mm) at the beginning of the distal third of the bone (Fig. 5a3). Left maxilla as long as ramus of premaxilla, head with robust neurocraniad process, remainder of bone with straight anterior but expanded posterior margin, the latter with marked dorsal wing. Right dentary preserved in medial view, robust; lower arm probably of same length as upper arm, but deeper. Teeth of dentary comprise at least three large canines (length 0.6–0.7 mm) in the anterior part and several smaller (length 0.2–0.5 mm) unicuspid teeth lying medial to the larger teeth up to the distalmost quarter of the bone (Figs. 5a3, c). The enlarged canines on the anterior tip of the premaxilla and the dentary represent outer row teeth and the smaller unicuspid teeth in between and medial to these represent the inner row teeth.

Anguloarticular slender-triangular, 1.24x longer than deep, posterior margin with small facet for lateral condyle of quadrate, pointed dorsal process. Retroarticular rather small and triangular and preserved in anatomical connection (Figs. 5a1–3).

#### Suspensorium and Opercular apparatus

The suspensorial bones are incompletely preserved. Palatine robust and bent, ventrally associated with small, slender ectopterygoid (Figs. 5a1–3). Hyomandibula with long and robust ventral process, large dorsally directed articulation facets; best visible in the Micro-CT data. Opercle crushed, probably relatively large, triangular. Of the subopercle only a long and pointed subopercular process is recognizable based on the Micro-CT sections, and runs parallel to the anteroventral margin of the opercle (Figs. 5a1–2). Interoperculum not preserved. Preopercle (?) robust, crescent-shaped, at least three lateral-line tubules recognizable ventrally; horizontal limb broad; vertical limb incomplete, but probably narrower. It is not absolutely clear whether this bone actually represents the preopercle. Due to the presence of lateral-line tubules, it could also be the second lacrimal but, judging from its position, it is more likely to correspond to the preopercle.

#### Hyoid and branchial arches

Anterior portion of left and right hyoid bars including the dorsal (?) and ventral hypohyals partly preserved; the border between the dorsal and ventral hypohyal and where they meet the anterior ceratohyal is not clearly visible. Ventral hypohyals robust, bearing a posteroventrally directed spine. Anterior ceratohyal abruptly becoming more slender towards the midline (Figs. 5a1–3). Basihyal triangular, recognizable between ceratohyals and dentary. Five branchiostegal rays can be discerned on the right side and at least two are visible on the left (Figs. 4, 5a1–3). Pharyngeal teeth bicuspid (with prominent cusp and shoulder), mostly slender, interspersed with broader bicuspid teeth (with one prominent and one minor cusp) (marked with an arrow in Fig. 5a1).

#### Vertebral column

Vertebral column slightly concave in the caudal region, 33 vertebrae, 19 abdominal and 14 caudal (Fig. 4, Table 1). Vertebral centra higher than long, hourglass-shaped, first and penultimate centra shorter than all others. Neural spines increasing in length from anterior to posterior with spines of last abdominal to first three caudal vertebrae longest, decreasing in length towards the caudal fin. Haemal spine of first caudal vertebra located posterior to third anal fin pterygiophore (Fig. 4). Fourteen pairs of long and slender ribs, first pair on fifth vertebra, parapophyses increasing in length posteriorly. No epipleurals recognizable. Supraneural bone club shaped (Figs. 4, 5a3).

#### Pectoral girdle and fins

Plate-like bone probably representing supracleithrum present underneath vertebral column (6th vertebra); cleithrum partially preserved, with ventral part pointed (both sides present), dorsal part probably missing; scapula not preserved; coracoid partially preserved in front of cleithrum; no postcleithrum; no pectoral fin discernible (Figs. 4, 5a3). Left posttemporal forked with robust dorsal process, ventral process seems more slender, but anteriorly broken. Probable dorsal process of right posttemporal preserved dorsally to left bone (Fig. 5a).

#### Pelvic girdle and fins

Basipterygia elongate, triangular plates that broaden posteriorly. Each pelvic fin with one strong spine and five branched, segmented rays, probably not reaching anal fin origin (Figs. 4, 5a1–3).

#### Dorsal fin

Dorsal fin continuous, 14 spines and 10 branched, segmented rays. Spines increase in length posteriorly. Rays do not reach posterior margin of hypural plates. 22 stout pterygiophores (last one supporting two rays), decreasing in length posteriorly; pterygiophore of last fin spine inserts behind neural spine of 17th vertebra. Pterygiophore of sixth ray thickened (Fig. 4).

#### Anal fin

Anal fin with 3 strong spines and 9 branched, segmented rays. Spines gradually increase in length. Rays reach the first third of caudal peduncle. Twelve pterygiophores in total, decreasing in size posteriorly; anteriormost three pterygiophores insert before last abdominal vertebra (Fig. 4). First two pterygiophores are fused, but their size proportions differ from that seen in recent cichlids as the first pterygiophore is longer than the second one, rather than shorter. Association between further spines/rays and pterygiophores unclear.

#### Caudal skeleton and fin

**Fig. 6:**
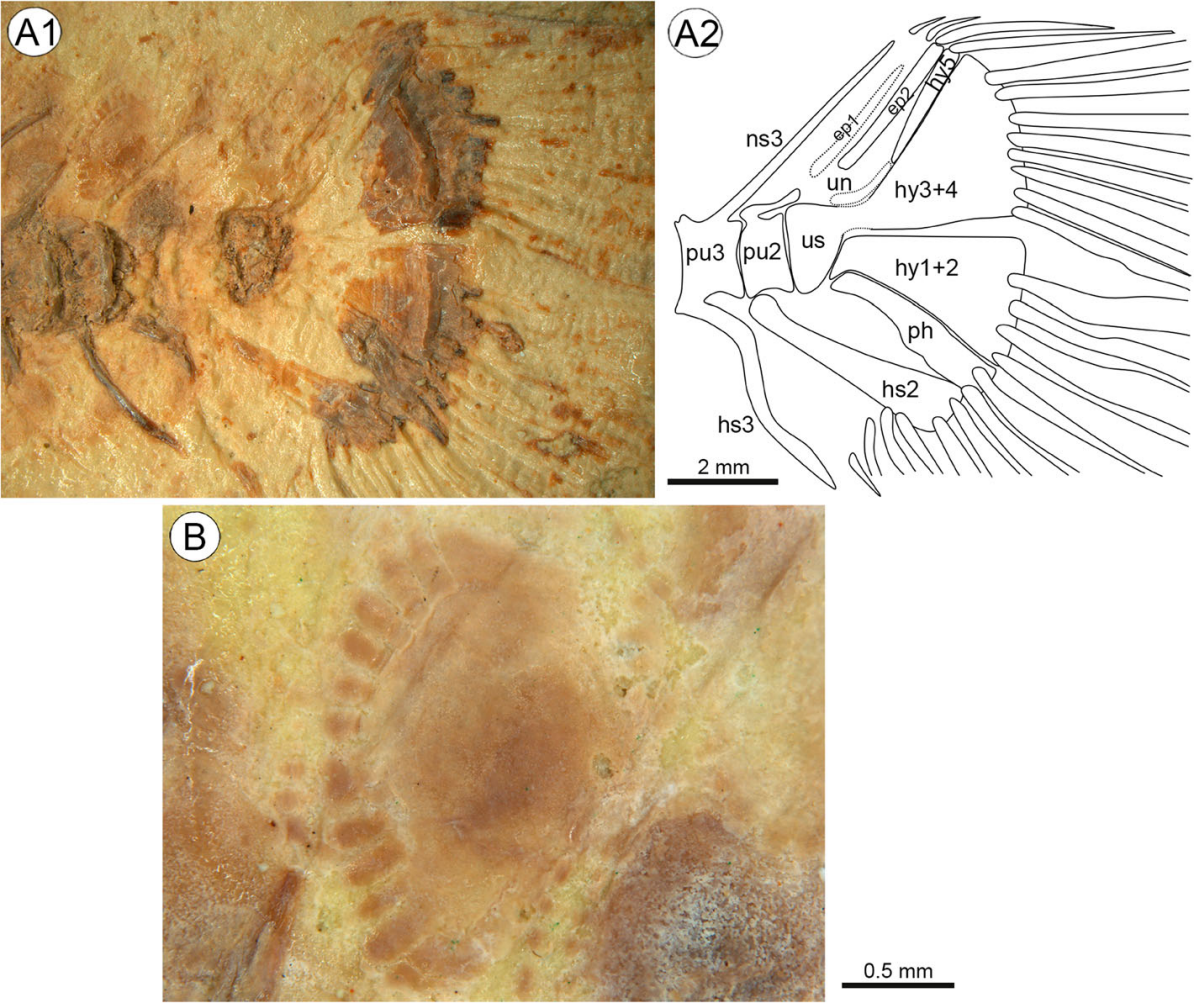
**A**, Caudal fin endoskeleton of † *W. unicuspidatus* gen. et sp. nov. based on light microscopy (**a1**) and interpretative drawing (**a2**); **b**, Close-up of scale (medial view) on dorsal part of caudal peduncle; note that rostral scale field is covered by neural spine. Photographs by first author. Abbreviations: **ep1, ep2**, epurals; **hs**, haemal spines; **hy1–5**, hypural plates; **ns**, neural spines; **ph**, parhypural; **pu**, preural centrum; **un**, uroneural; **us**, terminal centrum

The caudal axial skeleton comprises two broad hypural plates; hypural 1 + 2 is autogenous, hypural 3 + 4 is probably fused to the terminal centrum (urostyle). Hypural plates 1 + 2 and 3 + 4 show crests in their posterior parts (Fig. 6a1). A small and slender hypural plate 5 is positioned between hypural plate 3 + 4 and epural 2; it appears to reach the tip of the uroneural. Broad parhypural, located close to hypural 1 + 2. Two epurals, but only epural 2 is clearly discernible. Uroneural not distinctly visible, but probably above urostyle and proximal to hypural plate 5. Neural and haemal spine of preural centrum 3 supporting procurrent rays. Haemal spine of preural centrum 2 autogenous and broad; neural spine of preural centrum 2 probably absent (Fig. 6a).

Caudal fin slightly truncate (Fig. 4a1–2). It consists of 16 (8 + 8) segmented principal fin rays, of which the upper and lowermost are unbranched. The principal fin rays are supported by the parhypural, hypural plates 1 + 2 and 3 + 4. Four dorsal and five ventral procurrent rays are present.

#### Squamation

Scales preserved in medial view; scale type cycloid. Circuli mostly absent due to irregular granulation, which covers almost the entire scale (including focus; Fig. 6b), especially on flank scales; best recognizable on lateral fields.

Scales cover the whole body except the predorsal part; no scales on head. Approximately 28 scales in longitudinal line. Five or six? scale rows above vertebral column and about eight rows below. Divided lateral line, anterior lateral line about two scale rows below soft-rayed part of dorsal fin (Fig. 4a2) and two scale rows above body axis (= scale row bearing posterior lateral line according to Takahashi, [13]); no overlap (gap of two scales) between anterior and posterior lateral line; lateral-line canals clearly recognizable in one scale of the anterior and three scales of the posterior lateral line (arrows in Fig. 4a2); estimated total number of posterior lateral-line scales is at least seven.

Scale shape below dorsal fin and on dorsal part of caudal peduncle trapezoidal and wider than long (2.2 mm width and 1.6–1.7 mm length; width/length ratio 1.3–1.4) (Fig. 6b). Ventral scales posterior to anal fin more rounded (1.1 mm length and 0.9 mm width). Belly scales not recognizable. Around 11–13 broad and short radii (Fig. 6b).

## Discussion

### The best-fit approach

In the following, we justify the assignment of †*Warilochromis* to the pseudocrenilabrine tribes based on application of the ‘best-fit approach’ [76]. This method is similar to the established taxonomic assignment of fossil taxa, but, compared to previous works, uses a much more comprehensive dataset of extant species to dissect the phylogenetic affinities of the fossil.

**Fig. 7:**
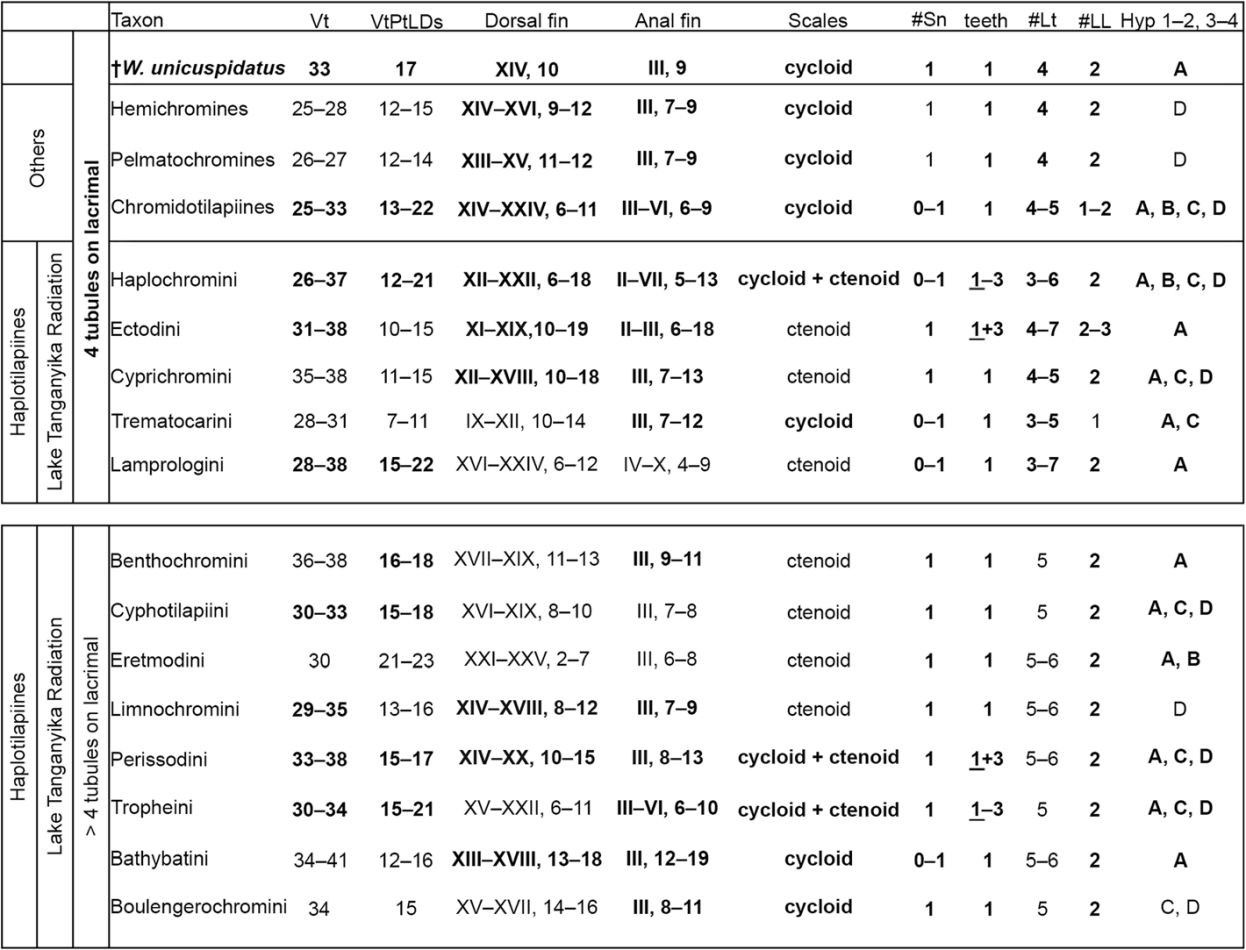
Morphological characters of †*Warilochromis unicuspidatus* gen. et sp. nov. and of all modern species of the tribes of the Lake Tanganyika Radiation and three further tribes of the Pseudocrenilabrinae in which the character combination single supraneural + exclusive unicuspid oral dentition (as seen in the fossil) can occur. Abbreviations: **Vt**, total number of vertebrae; **VtPtLDs**, ordinal number (s) of vertebrae associated with the pterygiophore of the last dorsal fin spine; **scales**, predominant scale type on flanks; **#Sn**, number of supraneural bones; **teeth**, inner and outer row teeth of oral dentition coded as follows: 1 = unicuspid, 2 = bicuspid, 3 = tricuspid, and underlining is used to indicate that exclusively unicuspid dentition is present in some species; **#Lt**, number of lateral-line tubules on the lacrimal; **#LL**, number of lateral-line segments on the body; **Hyp 1–4**, configuration of the hypural plates coded as follows: A = hyp 1 + 2, 3 + 4; B = hyp 1 + 2, 3, 4; C = hyp 1, 2, 3 + 4; D = hyp 1, 2, 3, 4. Source of data: comparative material (see Additional file 1: Supplementary Data S1 and Additional file 2: Supplementary Data S2) and literature: [89–189]

The study of our comparative dataset and related published data (e.g., [13, 85, 87, 88]) reveals that the character combination ‘single supraneural + exclusively unicuspid oral dentition + a lacrimal with four lateral-line tubules’ found in †*Warilochromis* occurs in eight tribes of extant African cichlids – namely Hemichromines, Pelmatochromines, Chromidotilapiines and five tribes of the haplotilapiines, i.e. the Trematocarini, Lamprologini, Cyprichromini, Ectodini and Haplochromini, the latter five belong to the Lake Tanganyika Radiation (Fig. 7; see also Fig. 1).

We argue that †*Warilochromis* cannot be allocated to the Hemichromines, Pelmatochromines or Chromidotilapiines for the following reasons. (i) †*Warilochromis* has fused hypural plates (Fig. 6a), and no such fusion is known for species of the Hemichromines or Pelmatochromines (our own data and [78]); and (ii) the shape of the crowns of the outer teeth as seen in labial view in †*Warilochromis* (Figs. 5b–c) is unlike that reported for the Chromidotilapiines, in which the outer-row teeth have ‘a unilaterally compressed cone, the compression being manifest on the labial aspect of the crown’ (Greenwood, [87]:158).

Among the five Lake Tanganyika tribes under consideration, we regard †*Warilochromis* as being clearly distinct from the Trematocarini and Lamprologini. The Trematocarini do not have a divided lateral line (their posterior lateral-line segment is absent), whereas a divided lateral line is present in †*Warilochromis* (see Fig. 4a2). In addition, Trematocarini are unique insofar as the pores of the lateral-line tubules on the lacrimal are distinctly larger than the tubule itself, such that adjacent pores are almost in contact (Takahashi, [88]:13–14, Fig. 7). The common condition in cichlids is that the diameter of the sensory pore corresponds to of the lateral-line tubule with which it is associated. In †*Warilochromis* the distal portion of the lateral-line tubules on the lacrimal is clearly discernible and adjacent tubules are widely separated from each other (Figs. 5a1–3). This indicates that the pores must have been further apart than in the Trematocarini, and of ‘normal’ size. Both features suggest that the new fossil does not exhibit the condition characteristic of the Trematocarini. Finally, †*Warilochromis* possesses only three anal fin spines (Fig. 4), and therefore differs from all members of the Lamprologini, which have more than three of these elements (Stiassny, [190]).

The three remaining candidate tribes to which †*Warilochromis* could belong based on this comparative approach are the Cyprichromini, Ectodini and Haplochromini. They share the following suite of features with †*Warilochromis*: a single supraneural bone, exclusively unicuspid oral dentition with normally shaped crowns, lacrimal with four lateral-line tubules, posterior lateral-line segment present, three anal fin spines and hypural plates 1 + 2 and 3 + 4 fused (see Fig. 7).

Among the character combination that defines the **Cyprichromini** are the following traits: fusiform elongated body (4 to 4.65x longer than high); minute and subconical pharyngeal teeth forming a comb-like row on the posterior border, with the rest of the pharyngeal bone being sparsely dentigerous; an emarginate caudal fin; strongly ctenoid scales; and 35–36 (17 + 18–19) vertebrae [12]. According to Takahashi’s [13] work, the members of this tribe can be recognized by the presence of the ‘neurocranial lateral-line foramen 0’ that is ‘separated from its opposite member’, a ‘forked caudal fin, and ctenoid scales at mid-body’ (Takahashi, [13]:377). It is unlikely that †*Warilochromis* represents a member of the Cyprichromini in light of its more compact body proportions (3.8x longer than high), bicuspid pharyngeal teeth that seem to have been evenly distributed on the pharyngeal jaws, slightly truncate caudal fin, cycloid scales, and fewer vertebrae (33 (19 + 14)).

The character combination that defines the tribe **Ectodini** includes the presence of exclusively ctenoid scales and an emarginate caudal fin [12], as well as a unique morphology of the palatine (with the posterior and dorsal margins forming a 90° angle) [191, 192]. In addition, Takahashi [13] proposed as synapomorphies for the Ectodini the presence of infraorbitals of type B (IO1 posteriorly elongated, opening through 4–7 pores, IO2–4 continuous, IO2 with 4–5 pores, IO3 + IO4 short; see [88] for details) and a palatopterygoid gap. Clearly, given its cycloid scales, truncate caudal fin and palatine morphology, †*Warilochromis* cannot be a member of the Ectodini.

Poll [12] defined the **Haplochromini** as having a rounded or subtruncate caudal fin; at least partially cycloid scales; bicuspid (or partially unicuspid) outer-row and tricuspid inner oral dentition; bicuspid, conical or molariform pharyngeal teeth; a dorsal fin with 13–20 spines and 8–11 rays; an anal fin with 3–6 spines and 7–10 rays; and the presence of 28–35 scales in the longitudinal line. Takahashi [13] revised this diagnosis and proposed the combination of type A infraorbitals (= composed of six elements, lateral line on lacrimal (IO1) branched into five tubules; sensu Takahashi, [88]); bicuspid outer and tricuspid inner teeth on both jaws; and ctenoid scales at midbody as diagnostic for this tribe. However, it should be noted that exceptions to several of these characters are known. (i) Not all species of the riverine genus *Pseudocrenilabrus* display five lateral-line tubules on the lacrimal: the number can vary from 3 to 6, with a modal number of 4 [127]; (ii) piscivorous species of *Haplochromis* in Lake Victoria and the riverine Serranochromines have unicuspid inner- and outer-row oral teeth (e.g., [41, 128, 193, 194]); (iii) the riverine genus *Haplochromis vanheusdeni* at least partially displays cycloid scales on the flanks (see [195]), which is also known for species of *Orthochromis* and the Serranochromines (e.g., [113, 128, 196]).

†*Warilochromis* is very similar to the Haplochromini if the character combination of the tribe as a whole (i.e. including the above-mentioned exceptions) is considered. It shares the morphology of the caudal fin, scales, oral teeth (of piscivores) and pharyngeal teeth. In addition, its meristics and also the number of lateral-line tubules on the lacrimal fall within the range of the Haplochromini (see Fig. 7). A close affinity with the Haplochromini is additionally supported by the results of our multivariate analysis (Additional file 6: Supplementary Fig. S2), which places †*Warilochromis* near to the center of the 95% ellipse of the Haplochromini. In contrast, there is only marginal overlap between †*Warilochromis* and the 95% ellipses of the Tropheini, Perissodini, Cyprichromini and Lamprologini.

However, none of the lineages currently included in the Haplochromini displays exactly the same character set as is present in †*Warilochromis* (see Table 2). For example, a lacrimal with the lateral line divided into four tubules is known only in species of the riverine genus *Pseudocrenilabrus* [21, 127]. But, unlike †*Warilochromis*, species of *Pseudocrenilabrus* display weakly ctenoid scales below the anterior lateral-line segment [127] and the outer row of their oral dentition is never exclusively unicuspid [127]. In addition, the total vertebral counts of *Pseudocrenilabrus* do not exceed 30 ([127] and this study), whereas †*Warilochromis* has 33.

**Table 2:** Character combination of the haplochromine lineages recognized in Schedel et al., [17] and the respective combination in †*Warilochromis* gen. nov

Taxon	Four lateral-line tubules on the lacrimal	Exclusive unicuspid oral dentition	Cycloid scales on flanks
**†*Warilochromis*gen. nov**	**+**	**+**	**+**
Lake Malawi flock	–	+/−	+/−
Riverine + Modern Haplochromini	–	+/−	–
Tropheini	–	+/−	+/−
*Haplochromis vanheusdeni*	–	–	+/−
*Astatoreochromis straeleni*	–	–	+/−
*Pseudocrenilabrus* group (incl. *Orthochromis, Pseudocrenilabrus*)	+/−	+/−	+/−
*Orthochromis indermauri*	–	–	+
Serranochromines	–	+	+/−
*Ctenochromis pectoralis*	–	–	–
Malagarasi *Orthochromis*	–	–	+/−

Information on morphological characters for recent haplochromine lineages from [12, 13, 21, 41, 88, 102, 113, 116, 119, 123, 127–129, 131, 148, 170, 171, 188, 193–216]. Abbreviations: **+**, present; **−**, absent

Taking all this information together, the particular combination of morphological characters exhibited by †*Warilochromis* clearly separates it from all other cichlid lineages except the Haplochromini, even though none of its included sublineages actually displays this precise set of traits. Thus, we propose that †*Warilochromis* represents an extinct genus and species whose affinities align it with the Haplochromini. Moreover, the peculiar configuration of the lacrimal with four lateral-line tubules argues for a close relationship with *Pseudocrenilabrus*, although some differences in squamation, dentition and meristics are evident (see above). The morphological differences between †*Warilochromis* and *Pseudocrenilabrus* could perhaps be explained by the fact that the extant members of *Pseudocrenilabrus* are adapted to a life in riverine habitats, as they are distributed in streams from Sudan to Uganda and the Congo Basin (e.g., [217]). In contrast, †*Warilochromis* derives from lake sediments.

### Comparison of †*Warilochromis* with previously described cichlid fossils from Africa, Arabia and Europe

We begin by comparing †*Warilochromis* with taxa for which the character states relating to oral dentition and/or number of lateral-line tubules on the lacrimal are known. Then we compare the new specimen with those fossils for which these characters are unknown. In each case, we indicate in the following how the listed taxon differs from †*Warilochromis*.

Taxa for which at least one of the characters ‘oral dentition’ and ‘number of lateral-line tubules on the lacrimal’ is known:

i. †*Mahengechromis* spp. Murray, 2000 [218]; middle Eocene (c. 46 MYA) of Tanzania: hooked (vs. conical) unicuspid oral teeth on the dentary; more dorsal fin spines (D XV vs. D XIV); fewer vertebrae (22–25 vs. 33)
ii. †*Macfadyena dabanensis* Van Couvering, 1982 [70]; Oligocene Upper Daban Series of Somalia: two supraneurals (vs. one); all hypural plates clearly separated from each other (vs. fused)
iii. †*Rhodopotilapia gracialis* Kirilova & Georgiev, 2015 [219]; early Oligocene Bulgaria: two supraneurals (vs. one); 27 vertebrae (12 + 15) vs. 33; D XIV,16 (vs. D XIV, 10); A III,11 (vs. A III, 9)
iv. †*Palaeofulu kuluensis* Van Couvering, 1982 [70]; lower to middle Miocene Kulu Fm (17–15 MYA) of Kenya: ‘leaf-shaped’ (vs. conical) unicuspid oral teeth; two supraneurals (vs. one); fewer vertebrae (25–29 vs. 33)
v. †*Oreochromimos kabchorensis* Penk et al., 2019 [76]; middle Miocene (12.5 MYA), site Kabchore, Ngorora Fm Member C: uni- and tricuspid oral teeth (vs. solely unicuspid); 28–30 vertebrae (vs. 33); hypural plates separated (vs. fused)
vi. †*Sarotherodon martyni* Van Couvering, 1982 [70]; middle Miocene (ca. 12 MYA), Kapkiamu Shales, Ngorora Fm Member C: few anterior tricuspid outer-row teeth (vs. solely unicuspid); 29 vertebrae (vs. 33); hypural plates separated (vs. fused)
vii. †*Rebekkachromis ngororus* and †*R. kiptalami* Kevrekidis, Valtl & Reichenbacher, 2019 [75]; uppermost middle to lowermost upper Miocene (ca. 11 MYA), site Rebekka, Ngorora Fm Member D: tricuspid (vs. unicuspid) oral teeth; two supraneurals (vs. one)
viii. †*Tugenchromis pickfordi* Altner, Schliewen, Penk & Reichenbacher, 2017 [16]; upper Miocene (ca. 9–10 MYA), site Waril, Ngorora Fm Member E: Six lateral-line tubules (vs. four) on the lacrimal; fewer vertebrae (29 vs. 33)
ix. Cichlidae indeterminate Van Couvering, 1982 [70]; upper Miocene Mpesida Beds (ca. 6.8 MYA): stout unequally tricuspid to conical unicuspid oral teeth (vs. solely unicuspid)
x. †*Oreochromis lorenzoi* Carnevale, Sorbini & Landini, 2003 [220]; upper Miocene Gessoso-Solfifera Fm (ca. 6 MYA): bicuspid and tricuspid oral teeth (vs. solely unicuspid); more dorsal and anal fin spines (D XV vs. D XIV and A IV vs. A III); deep-bodied
xi. cf. *Pelmatochromis* Van Couvering [70]; lower Miocene Lamitina beds (ca. 22 MYA) of Uganda: six lateral-line tubules (vs. four) on the lacrimal
xii. ‘Cichlidae indeterminate’ by Van Couvering [70]; lower Miocene Turkana Grits (17.5 ± 0.9–16.7 ± 0.8 MYA), Kenya: recurved, weakly tricuspid to sometimes unicuspid teeth and stout, equally bicuspid teeth (vs. solely unicuspid)
xiii. ‘Cichlidae indeterminate Form A’ by Van Couvering, [70]; lower to middle Miocene Kulu Fm (17–15 MYA) of Kenya: tricuspid outer and bicuspid inner oral teeth (vs. solely unicuspid)
xiv. ‘Cichlidae indeterminate Form B’ by Van Couvering [70]; lower to middle Miocene Kulu Fm (17–15 MYA) of Kenya: unequally bicuspid oral tooth (vs. solely unicuspid)
xv. ‘Cichlidae indeterminate–Group A’ by Van Couvering [70]; middle Miocene Kirimun Beds Kenya: unicuspid, bicuspid and tricuspid oral teeth (vs. solely unicuspid)

List of fossil African cichlid taxa for which the characters ‘oral dentition’ and ‘number of lateral-line tubules on the lacrimal’ are unknown:

i. cf. *Tylochromis* Otero et al. [221] and Murray [222]; middle Eocene (37–39 MYA) of Libya and the upper Eocene-lower Oligocene (35.1–33.8 MYA) of Egypt ([221, 222]): molariform pharyngeal teeth (vs. bicuspid)
ii. ‘Cichlidae indeterminate Form B’ by Van Couvering [70]; Oligocene Upper Daban Series of Somalia: dorsal fin with D? XI,13 (vs. D XIV,10); 22 scales in longitudinal line (vs. approx. 28); hypural plates separated (vs. fused)
iii. ‘Cichlidae indeterminate Form D’ by Van Couvering [70]; Oligocene Upper Daban Series of Somalia: fewer vertebrae (24 vs. 33); D XIII,11 (vs. D XIV,10); A VI,12 (vs. A III,9)
iv. Unnamed fossil by Weiler [223]; Oligocene–Miocene of Jordan: fewer vertebrae (23 vs. 33)
v. ?*Heterochromis* Lippitsch & Micklich [224]; Lower Miocene sediments of the Baid Fm of SW Saudi Arabia: probably 2 supraneurals (vs. 1); D XIII–XIV,13+ (vs. D XIV,10); AIV–V (vs. A III)
vi. Second species (unnamed) of Lippitsch & Micklich [224]; lower Miocene sediments of the Baid Fm of SW Saudi Arabia: scaly soft-rayed part of the dorsal fin (vs. scaleless)
vii. ‘Cichlidae indeterminate–Group B’ by Van Couvering [70]; middle Miocene Kirimun Beds Kenya: anal fin extending to origin of caudal fin skeleton (vs. not); caudal fin with five hypurals (vs. fused)
viii. Unnamed cichlids by Argyriou [225]; upper Miocene sediments (ca. 6.8 MYA) of the Sahabi Fm from Sahabi, Libya: anterior process of the anguloarticular broad (vs. slender), ventral process of this bone short and almost vertical to anterior one (vs. acute angle between anterior and ventral processes).

### Previously described fossils putatively assigned to the Haplochromini

According to Van Couvering [70] and Lippitsch & Micklich [224], six fossil taxa (not included in the lists above) can be putatively attributed to the Haplochromini because they share some important features with them. However, all but one exhibit characters that are not typical for the Haplochromini (see below).

i. ‘Cichlidae indeterminate Form A’, Van Couvering [70]; Oligocene Upper Daban Series of Somalia: These isolated bones were attributed to a single species closely related to the haplochromines, perhaps intermediate between *Hemichromis* and *Haplochromis*, based on the presence of weakly tricuspid pharyngeal teeth. However, tricuspid pharyngeal teeth are also known from species of *Coptodon* (e.g., [226]) and South American cichlids (e.g., [227, 228]), and also occur in other fish families/orders e.g., Cyprinidae [229]; Hemiramphidae [230]; Cyprinodontiformes [231]. In fact, the striking shape of these teeth closely resembles that of the pharyngeal teeth of piscivorous Haplochromini [232], *Coptodon* (tribe Coptodonini) [226, 233], and Cichlinae of the genera *Geophagus* [228] and *Apistogramma* [227]. In addition to pharyngeal teeth, Van Couvering [70] described granular cycloid scales, which could indicate a relationship to the Tropheini. However, without inspection of the original material, the assignment of these isolated bones remains uncertain.
ii. ‘Cichlidae indeterminate Form C’, Van Couvering [70]; Oligocene Upper Daban Series of Somalia: Van Couvering [70] tentatively places the two partially preserved articulated skeletons (probably counterparts) among the haplochromines, because of the presence of four anal fin spines and ctenoid scales. However, the author mentions that two supraneurals could be discerned, and this condition is not found in the Haplochromini (see Fig. 7). The character combination displayed by these fossils (ctenoid scales + > 3 anal fin spines + two supraneurals) is otherwise known only among present-day South American Cichlinae (e.g., [79, 234, 235]) and any relationship with the haplotilapiines or Haplochromini can be excluded.
iii. †*Kalyptochromis hamulodentis* Van Couvering, 1982 [70]; lower to middle Miocene Kulu Fm (17–15 MYA) of Kenya: Van Couvering indicates that this fossil species shares with the haplochromine genera *Tropheus* and *Pseudotropheus* the bicuspid oral dentition, polyacanthous median fins and a large number of vertebrae. But she tentatively placed it near the Etroplines and the Tilapiines because she also noted the presence of some plesiomorph characters such as (i) two supraneurals, (ii) seven or more branchiostegal rays, (iii) a long hyoid complex and (iv) a hyomandibula with a short body, short ventral process, and large anteroventral process. We consider a close relationship with the Etroplinae to be unlikely, because the latter possess tricuspid or unicuspid teeth (see [236]). Among the remaining cichlids the combination of two supraneurals, four anal fin spines and bicuspid oral dentition seen in †*Kalyptochromis* is only known among the Tilapiini and Oreochromini (see [15, 76]). However, even an assignment to the Cichlidae is problematic, because cichlids (by definition) have no more than five branchiostegal rays.
iv. †*Nderechromis cichloides* Van Couvering, 1982 [70]; lower to middle Miocene Kulu Fm (17–15 MYA) of Kenya: Van Couvering putatively attributed this fossil species to the haplochromines because of the morphology of its pharyngeal apophysis and parasphenoid, and the presence of ctenoid scales. However, the combination of a *Haplochromis*-type pharyngeal apophysis and ctenoid scales is also known for the South American genus *Cichla* [237] as well as for members of the Ectodini, Eretmodini, and Lamprologini (e.g.,) [238–240]. Moreover, the outline of the parasphenoid is difficult to discern in the figures in Van Couvering’s [70] paper. According to her interpretation of a conical oral dentition that lacks inner teeth, this fossil could be related to the Ectodini, or Eretmodini (see [13]).
v. Third species (unnamed) of Lippitsch & Micklich [224]; lower Miocene sediments of the Baid Fm of SW Saudi Arabia: Lippitsch and Micklich [224] putatively assigned two fossil specimens from the Baid Fm to the haplochromines based on their combination of (i) ctenoid scales; (ii) anal fin with (probably) four spines and at least 10 rays; (iii) probably separated hypural plates. In addition, in the first description of the material by Micklich and Roscher [241], the authors mentioned the presence of probably numerous (in several rows), small (< 1 mm), slender, slightly recurved, oral teeth, of which some might have been unicuspid. Among the extant Haplochromini and Tropheini, only *Tropheus*, *Astatoreochromis*, *Orthochromis torrenticola* and *O. machadoi* possess so many anal fin spines (this study and [12, 13, 94, 113, 126, 199]). All of them have a mixture of uni-, bi-, and tricuspid dentition [12, 94, 113, 126, 198, 216], and a combination of ctenoid and cycloid body scales [13, 126, 216]. Thus, a phylogenetic relationship between this fossil and the Haplochromini seems possible.
vi. †*Palaeochromis rouselleti* and †*P. darestei* Sauvage, 1907 [242]; upper Miocene Seybouse Gypsiferous Marls (> 7 MYA) of Algeria: Based on its leaf-shaped unicuspid, unequally bicuspid or tricuspid oral, and hooked bicuspid pharyngeal teeth, Van Couvering [70] puts †*Palaeochromis* in an intermediate position between *Pelmatochromis* sensu lato (sensu Thys van den Audenaerde [243]) and *Tilapia* sensu stricto (=Tilapiini without *Chilochromis*, see [15]). She also noted that *Pseudocrenilabrus* and some generalized species of *Haplochromis* display similar oral and pharyngeal tooth morphology, but that †*Palaeochromis* differs from both genera in its cycloid rather than ctenoid scales. However, there is an inconsistency in the generic diagnosis of †*Palaeochromis*, because Van Couvering [70] states that the scales are cycloid or ctenoid in †*P. rouselleti*, while in the generic diagnosis for †*Palaeochromis* she mentions only granular cycloid scales. In addition, the assumption that all species of *Pseudocrenilabrus* and *Haplochromis* have ctenoid scales is incorrect, because some members of both genera can display both ctenoid as well as cycloid body scales (e.g., [127, 187, 194, 196]). Leaf-shaped teeth are typical for scale-eating cichlids of the tribe Perissodini (e.g., [244]), but have not been described for Haplochromini (e.g., [196]).

In summary, of the previously described putative haplochromine fossil cichlids, only the ‘third species (unnamed)’ of Lippitsch & Micklich [224] from the Lower Miocene sediments of the Baid Fm of SW Saudi Arabia can be reasonably attributed to the Haplochromini.

In addition, each of the taxa discussed above are clearly distinct from our fossil. †*Warilochromis* does not show the tricuspid pharyngeal teeth seen in ‘Cichlidae indeterminate Form A’. The taxon ‘Cichlidae indeterminate Form C’ has a very different combination of characters (see above) from that seen in †*Warilochromis*. The same is true for †*Kalyptochromis hamulodentis*, which has bicuspid dentition, two supraneurals, seven or more branchiostegal rays, more dorsal and anal fin spines (D XVII vs. D XIV and A IV vs. A III), and fewer vertebrae (30 vs. 33). In addition, there is a clear difference between †*Nderechromis cichloides* and †*Warilochromis* due to the presence in the former of ctenoid (vs. cycloid) scales and the lack of oral inner teeth (vs. present). The ‘third species (unnamed)’ of Lippitsch & Micklich [224] differs from †*Warilochromis* in having ctenoid scales, probably four anal fin spines and probably separated hypural plates. Finally, †*Palaeochromis rouselleti* and †*P. darestei* are clearly different from †*Warilochromis* because of their distinctive dentition, as described above.

#### Palaeoecology of †Warilochromis

The new fossil taxon possesses fang-like caniniform teeth, which is a typical feature of predatory fish (e.g., [245]). The fact that only a single specimen of †*Warilochromis* was found among the material recovered from the Waril site further supports this interpretation, as predators are expected to occur at much lower levels than their prey (e.g., [246–248]).

Among African cichlids, predatory species are known from almost all tribes and lineages. The most detailed functional classification of predatory strategies has been proposed by Schmitz [249]. He divides them into three categories: ambush (sit-and-wait); ballistic capture (sit-and-pursue); and pursuit hunters, which actively seek and chase their prey. This last type of hunting strategy requires a streamlined body shape adapted to rapid swimming, whereas the other two strategies do not (see [245]). Given its relatively compact body shape, †*Warilochromis* may have been either a sit-and-pursue or sit-and-wait hunter.

The sit-and-pursue strategy is relatively common among extant cichlids, and has been described for *Cichlasoma* (e.g., [250]), *Crenicichla* (e.g., [251]), *Hemichromis* (e.g., [252]), many Lamprologini (e.g., [253, 254]), Perissodini (e.g., [255]) and Haplochromini (e.g., [253, 256, 257]). In contrast, among African cichlids, the sit-and-wait strategy is only known for the piscivorous genus *Nimbochromis* of the Lake Malawi Haplochromini. This fish lies on the bottom on its side, and looks like a dead fish, owing to its blotchy coloration [37, 258, 259]. As soon as a scavenger gets close enough, the predator engulfs it [37, 258–260]. This strategy is also known as thanatosis or death feigning [37]. It was recently described also for a South American cichlid [259].

The only previously described putative haplochromine fossil (=third species (unnamed) of Lippitsch & Micklich, [224]) has unicuspid oral teeth according to the original description, but further differentiation was hampered by their poor preservation (see [241]). If its oral dentition was exclusively unicuspid, this lower Miocene species may also have had a predatory lifestyle.

#### New insights into the evolutionary history of the Lake Tanganyika Radiation cichlids

**Fig. 8:**
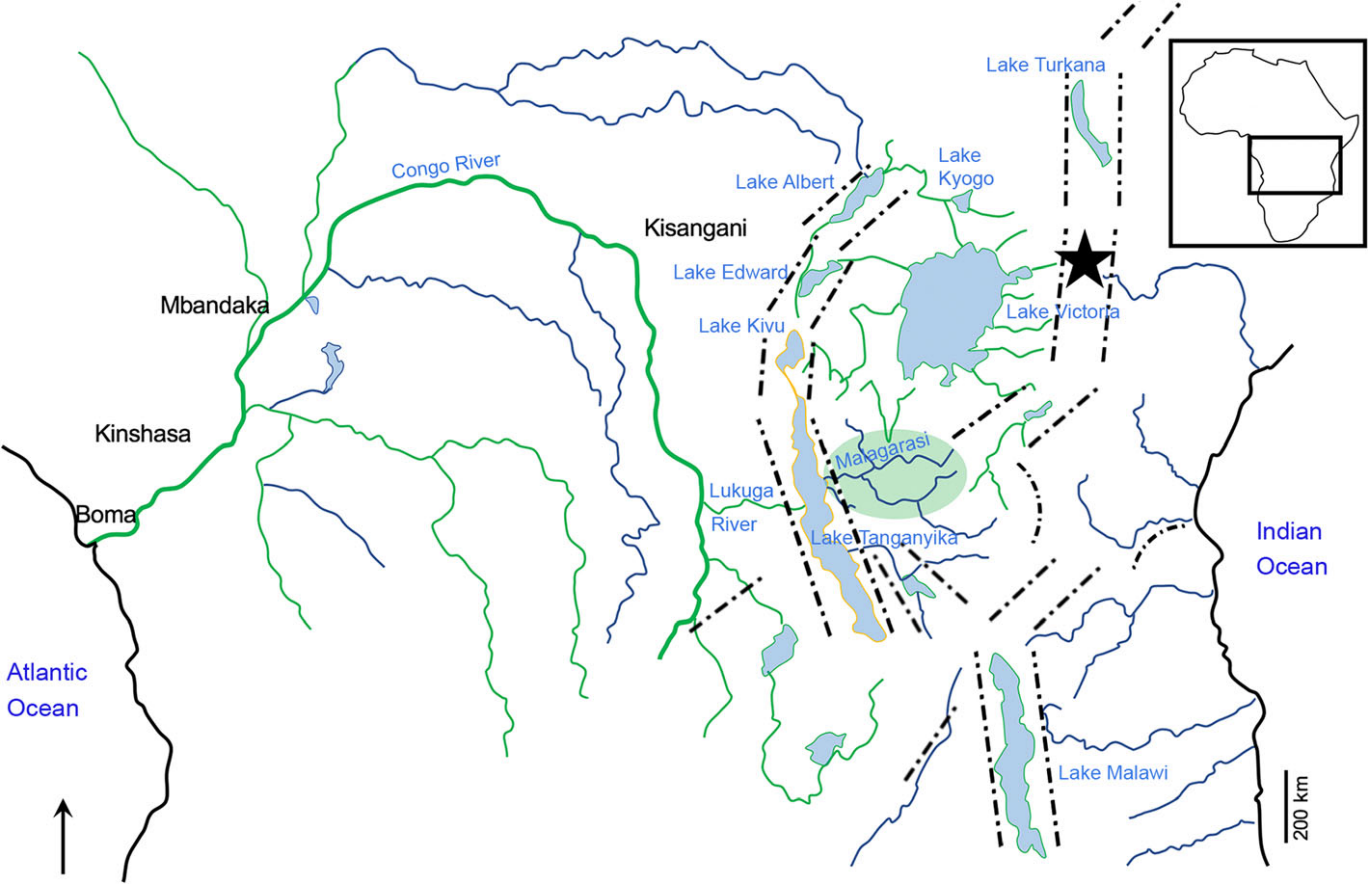
Map of East and Central Africa depicting the main present-day lakes and rivers, the tectonic structures of the Western and Eastern branches of the East African Rift System (dash-dotted lines) and the location of Palaeolake Waril (star), where the new fossil cichlid was found. The distribution of the present-day members of the Haplochromini in Lake Tanganyika (orange line), Lake Malawi and Victoria (green line), adjacent river systems and the Congo and Malagarasi Rivers is shown (green shading and lines). Source of map: River network drawn after Clabaut et al. [261] and Schwarzer et al. [262], distribution of haplochromine cichlids according to Schwarzer et al. [24]

Here we have shown that †*Warilochromis* can be assigned to the Haplochromini (Fig. 7) and could be related to *Pseudocrenilabrus* (Table 2). Its age and the site of its discovery in the Central Kenya Rift, which forms part of the eastern branch of the East African Rift System and is thus quite remote from Lake Tanganyika (Fig. 8), make it particularly noteworthy for a better understanding of the evolutionary history of the Haplochromini.

Salzburger et al. [25] introduced the notion of a ‘primary lacustrine radiation’ to explain the peculiar branching pattern seen among the Lake Tanganyika cichlids, i.e. the occurrence of several lineages separated by short branch lengths in sequence-based phylogenetic trees, as also revealed by other studies [28, 263, 264]. This pattern could result from a shift in diversification rates owing to the advent of fully lacustrine conditions in Lake Tanganyika around 5–6 MYA [25]. Salzburger et al. [25] recognized two series of cladogenetic events. The first series probably happened during the ‘protolakes’ stage (see below) and involved six seeding lineages (the ancestors of *Boulengerochromis*, Trematocarini, Bathybatini, Lamprologini, Eretmodini and the C-lineage (sensu Clabaut et al., [261]; including the tribes Benthochromini, Cyprichromini, Cyphotilapiini, Ectodini, Haplochromini, Limnochromini and Perissodini). The second series likely represents the primary radiation that took place in the fully lacustrine habitat and involved the major diversifications of the Lamprologini and the C-lineage (sensu Clabaut et al., [261]).

It is generally is assumed that the basin of Lake Tanganyika formed 9–12 MYA (see [265]). This is based on the work of Cohen et al. [266] who, in the absence of stratigraphic data for deposits older than 35 ka in the Lake Tanganyika Basin, used short-term sedimentation rates (derived from radiocarbon-dated core samples in Lake Tanganyika) and an approximate total sediment thickness for the Lake Tanganyika Basin (derived from reflection-seismic data) to calculate this age model. Moreover, a three-stage model of origin has been proposed for the Lake Tanganyika Basin by Tiercelin and Mondeguer [267]. These authors used significant signatures and changes in the seismic data and combined seismically derived sediment thicknesses in the Lake Tanganyika Basin with sedimentation rates drawn from the literature. Their three-stage model has been adopted and/or emended by subsequent authors (e.g., [265, 268]; see also [25, 269, 270]). According to the last group of authors, Stage 1 (20–14 MYA) was characterized by a weakly subsiding tectonic basin located in the western branch of the East African Rift System. It was drained by a wide, meandering stream and may have been connected to the Congo River drainage system. In Stage 2 (= ‘protolakes’ stage; 14–6 MYA), the development of tectonic half-grabens led to the progressive formation of several small, shallow (< 50 m) and mostly isolated lakes. During Stage 3 (6–5 MYA), the half grabens were gradually connected into a large fault structure, and the isolated shallow lakes were progressively replaced by a single large lake, with deep-water conditions emerging at about 5 MYA. It seems plausible that this formation of deep-water lake conditions about 5 MYA gave rise to an adaptive radiation of the cichlid fishes in Lake Tanganyika, and therefore this age is commonly used as a calibration constraint (minimum age) in attempts to trace the course of the Lake Tanganyika Radiation of the cichlids (e.g., [271], and references therein).

Most publications use the age estimate proposed by Cohen et al. [266] for the formation of Lake Tanganyika of about 9–12 MYA. However, other estimates also exist, ranging from 14.5 to 5.5 MYA (see [272–274]). Previous studies focusing on molecular phylogenetics and divergence times of the Cichlidae use as calibration for the evolution of the Lake Tanganyika cichlids either the 9–12 million years (m.y.) age, or the 5–6 m.y. age for the emergence of deep lacustrine conditions within Lake Tanganyika [22, 275, 276]. Depending on the calibration point used, the nodes of the Haplochromini and Tropheini have highly variable ages, with ranges from 23 to 3 MYA and 11–2 MYA, respectively (see Table 3). Schedel et al. [17] calculated an age of nearly 12 MYA for the node of the whole *Pseudocrenilabrus* group. The finding of †*Warilochromis*, with an age of 9–10 MYA makes node ages for the Haplochromini younger than 9 MYA, as suggested by Koblmüller et al. [22], Friedman et al. [277], Meyer et al. [278] and Irisarri et al. [27] (see Table 3), unlikely. In addition, the age of the fossil also refutes the ‘out of Tanganyika’ hypothesis (sensu Salzburger et al., [45]) and the postulated date of 5–6 MYA for the onset of the divergence of the Haplochromini.

**Table 3:** Summary of molecular studies dealing with the divergence times of cichlids with the respective calibration points used as well as the node age of the Haplochromini and Tropheini

Study/Reference	Calibration point	Node age (MYA; mean, SD or 95% confidence interval or 95% HPD interval or 95% credibility interval)	
		Haplochromini	Tropheini
Genner et al., 2007 [11]	Gondwana; mitochondrial DNA only	22.7 ± 3.5	6.8 ± 1.1
	Cichlid fossils; mitochondrial DNA only	10.5 ± 1.6	3.0 ± 0.5
Day et al., 2008 [275]	Root of LTR set to 12 MYA	–	3.4, 2.7–4.5
	Root of LTR set to 28 MYA based on calibrations of Gondwana fragmentation	–	7.9, 6.3–10.5
Koblmüller et al., 2008 [22]	Lacustrine habitat in Lake Tanganyika (5–6 MYA) and Lake Malawi (0.57–1 MYA); Age of Lake Victoria superflock (0.2 MYA)	5.3, 4.9–5.7	3.4, 3.0–4.0
Santini et al., 2009 [279]	43 non-cichlid fossils; †*Mahengechromis*, Oligocene *Heterochromis* (23.3 MYA)	Ca. 9	–
Friedman et al., 2013 [277]	10 non-cichlid fossils	Ca. 7	< 5
Meyer et al., 2017 [278]	Concatenation	–	–
	Multispecies coalescent model	Ca. 5	–
Irisarri, 2018 [27]	Calibration scheme C10:	6.9, 3.3–10.5	4.3, 1.9–6.7
	Gondwana fragmentation:		
	–Madagascar + India/Africa + South America max. 165 MYA		
	–Africa/ South America max. 101 MYA		
	– Madagascar/ India 88 MYA		
	– split Cichlasomatini/ Heroini 45.7–101 MYA		
	– Second split within African cichlids (excl. *Heterochromis*) 45.7–101 MYA		
	– Haplotilapiines (without Etiini) 9.3–62 MYA		
	– Oreochromini 5.98–47.5 MYA		
	– African cichlids 33.1–79.6 MYA		
	– H-lineage/ Lamprologini 9.3–43.2 MYA		
Schedel et al., 2019 [17]	Calibration set 4:	16.6, 14.3–19.2	8.7, 6.8–10.7
	– †*Tremembichthys* 55.8–23.0 MYA		
	– †*Gymnogeophagus eocenicus* 45.4–39.9 MYA		
	– †*Plesioheros chaulidus* 45.4–39.9 MYA		
	– †*Oreochromis lorenzoi* 7.24–5.33 MYA		
	– †*Tugenchromis pickfordi* 9–10 MYA		
	– Onset Lake Barombi Mbo 1.12–0.98 MYA		
	– Estimated divergence age for family Cichlidae by Matschiner et al. (2016) 82.2–98.9 MYA		

Abbreviations: *HPD* Highest posterior intervals, *LTR* Lake Tanganyika Radiation, *MYA* Million years ago, *SD* Standard deviation

The only other fossil species that could reasonably represent a haplochromine, i.e. the third species (unnamed) of Lippitsch & Micklich [224], derives from Lower Miocene sediments of the Baid Fm of SW Saudi Arabia. If its assignment to the Haplochromini can be confirmed in future work based on additional material, it would represent the oldest known fossil member of the Haplochromini. Furthermore, it would support the idea that the Haplochromini originated east of Lake Tanganyika, as proposed by Schedel et al. [17], as well as a scenario in which haplochromine cichlids were already present prior to the formation of Lake Tanganyika and colonized the lake only later. This would also confirm the conclusion of Altner et al. [16], based on †*Tugenchromis pickfordi* (which derives from the same locality as †*Warilochromis*), that ancient progenitors of the Lake Tanganyika Radiation were already present in the Miocene of the eastern branch of the East African Rift System (Fig. 8).

In addition, both †*Warilochromis* and †*Tugenchromis* support the hypothesis of a late Miocene hydrological connection between the Central Kenya Rift and Lake Tanganyika, and imply that ancient Lake Tanganyika cichlids were able to migrate through riverine waters during the late Miocene (Fig. 8). Such a hydrological network would be consistent with previous geological studies that proposed an ancient east-west hydrological connection between the Malagarasi and the Congo Rivers, which was probably disrupted by the rifting processes that led to the formation of Lake Tanganyika [265, 267, 280–282].

## Conclusion

The fossil cichlid described here as †*Warilochromis unicuspidatus* gen. et sp. nov. represents an extinct genus and species of the Haplochromini, possibly related to the extant genus *Pseudocrenilabrus*. The age of the new fossil (9–10 MYA) implies that the Haplochromini must have emerged before this time and rules out the ‘out of Tanganyika’ hypothesis [45], i.e. the assumption that the divergence of the Haplochromini began only 5–6 MYA [25].

†*Warilochromis* was discovered at the site Waril in Central Kenya, in the eastern branch of the East African Rift. Both †*Warilochromis* and the putatively oldest fossil haplochromine from Saudi Arabia described earlier [224] are compatible with the suggestion, based on molecular data [17], that the Haplochromini first evolved east of Lake Tanganyika. Moreover, the remote location where †*Warilochromis* was found, together with the recent distribution of the Haplochromini, supports a hydrological connection between the Central Kenya Rift and the Lake Tanganyika drainage system, as suggested earlier in the context of the finding of the fossil cichlid †*Tugenchromis pickfordi*, a putative stem lineage of Lake Tanganyika cichlids [16].

With its fang-like oral dentition, †*Warilochromis* probably exhibited predatory, hunting behavior. In the absence of securely assigned haplochromine fossils with a similar type of teeth, †*Warilochromis* represents the first fossil predatory haplochromine. Furthermore, it indicates that this lifestyle had already evolved by 9 MYA, and that Haplochromini were already an important component of the East African drainage systems at that time.

## Methods

### Fossil material

One complete specimen preserved in lateral view (2014-WA-16). The fossil specimen is currently housed at the Department of Earth and Environmental Sciences, Ludwig-Maximilians University Munich, and will be deposited in Kipsaraman, Baringo County, Kenya, as soon as the new Baringo County Geopark has been established.

### Comparative material representing recent species

The new fossil exhibits characters that can also be found in the Pelmatochromines, Hemichromines and Chromidotilapiines and in several tribes that contributed to the Lake Tanganyika Radiation. Consequently, the comparative dataset comprises all cichlid tribes and lineages of the Lake Tanganyika Radiation (see Fig. 1b) as compiled in Altner et al. [16], and newly assembled comparative data for the Pelmatochromines, Hemichromines and Chromidotilapiines (X-ray images of 42 species (14 genera, 94 individuals, see Additional file 1, Supplementary Data S1 and Additional file 2: Supplementary Data S2). For details concerning the degree of completeness of the dataset relating to the tribes and lineages of the Lake Tanganyika Radiation, see Altner et al. [16]: p. e1297819–5, and for similar information on the other datasets see Additional file 1: Supplementary Data S1.

### Fossil preparation, measurements, imaging

Sediment particles covering the fossil were removed under a stereoscopic microscope (Leica M165 C) using a fine carbide needle mounted on the end of a mechanical pencil barrel. The specimen was investigated using both optical microscopy and X-ray micro-CT scanning. Micrographs were taken with a Leica M170 HD camera mounted on the same microscope and merged in Adobe Photoshop using the ‘photomerge’ option. Measurements were performed based on the merged microphotographs in ImageJ 1.49a [283] and recorded to the nearest 0.1 mm. Measurements are shown in Additional file 3: Supplementary Fig. S1. All measurements were normalized with reference to the standard length. Graphical illustrations were prepared on the basis of the composite microphotographs.

The x-ray images of recent specimens were assembled with a Faxitron UltraFocus digital X-ray cabinet.

The micro-CT scanning was performed on a Phoenix Nanotom m (GE Sensing & Inspection Technologies GmbH). The anterior and posterior parts of the fossil (with an overlapping area) were scanned separately. 1200 (anterior part) and 1280 (posterior part) projections (2.5 s each) were taken at a voltage of 120 kV and a current of 130 mA. The resulting 16 bit data sets (voxel sizes: 18 and 19.59 μm) were histogram adjusted and converted to 8 bit. The data sets were co-registered manually and merged using Amira 6.4 (FEI, Hillboro, OR, USA). The tool Volume Rendering of Amira was applied to examine the data.

### Morphological studies (fossil and comparative material)

Abdominal vertebrae were identified by the absence of a closed haemal arch (according to Barel et al. [232]). Total vertebral counts include the terminal centrum (urostyle). In the dorsal and anal fins, every fin ray was counted regardless of whether it was supported by a pterygiophore or not. Interpretation of osteological characters follows Stiassny [85] and Takahashi [13, 88]. In total, the meristic counts cover nine characters, namely (i–iv) the numbers of all discernible dorsal and anal fin spines and rays, (v–vii) counts of abdominal, caudal and total vertebrae, (viii) the number of supraneural bones, and (ix) the ordinal number of the vertebra (counted anteriorly to posteriorly) that is associated with the pterygiophore of the last dorsal fin spine.

### Morphological character matrix

A morphological data matrix that includes all African cichlid lineages is currently not available for a phylogenetic analysis. Here we use the morphological matrix of Stiassny [85] to tentatively place the new fossil within the Cichlidae (see [76] for same approach). Stiassny’s [85] original matrix comprises 18 ingroup taxa and 28 characters (19 osteological; 8 soft tissue; 1 behavioral). We added her ‘generalized percomorph taxon’ as the outgroup (see [76] for details). We added the fossil taxon to the data matrix by inserting character states for four (of 28) characters (respective states given in brackets), i.e., numbers 8(0), 11(0), 25(1), 27(1); these characters refer to the suture between the vomerine wing and the parasphenoid, the total number of vertebrae, the number of supraneural bones and the number of tubules on the lacrimal (see Additional file 4: Supplementary Data S4 and Additional file 5: S5). The morphological matrix was edited in Mesquite 3.51 [284].

### Phylogenetic analysis

Phylogenetic analyses of the morphological data matrix was performed under maximum parsimony in TNT 1.1 [285], using a combination of ‘new technology’ search options, i.e., parsimony ratchet, tree-drifting and tree-fusing. We used implied weighting (K = 12.0) according to Goloboff et al. [286]. In all other cases, the preset default settings were used. Clade support was assessed using standard bootstrapping (1000 replicates, absolute frequencies values). Following Hillis and Bull [287], clades with bootstrap values ≥70% were considered well supported. Phylogenetic trees were visualized and edited in FigTree 1.4.4 [288].

### Statistical analysis

A principal coordinates analysis (PCoA) was run using PAST [289] version 3.18, in order to explore meristic-count variation between the new fossil taxon and the 16 modern pseudocrenilabrine tribes – in which the character combination single supraneural + exclusive unicuspid oral dentition (as seen in the fossil) can occur – on the basis of our comparative dataset (Additional file 2: Supplementary Data S2). The nine aforementioned meristic characters were used as variables.

## Supplementary information

**Additional file 1.** Supplementary Data S1. Comparative material of extant species used for X-ray analysis and assembly of meristic data.

**Additional file 2.** Supplementary Data S2. Meristic counts from x-rays.

**Additional file 3. **Supplementary Fig. S1. Morphometric measurements conducted for this study. **A**, generalized cichlid fish depicting head-, body-, and fin-related linear measurements (re-drawn based on de Zeeuw et al., 2010 [114], Fig. 14 distributed under CC-BY license (https://creativecommons.org/licenses/by/4.0/) with permission from Naturalis Biodiversity Center https://www.repository.naturalis.nl/record/358750). **B1–2**, Upper and lower jaw bones (right side, lateral view) of a haplochromine cichlid with measurements (modified from Van Oijen & de Zeeuw, 2008] [194]: Figs. 3 & 6., based on *Haplochromis vonlinnei,* distributed under CC-BY license (https://creativecommons.org/licenses/by/4.0/) with permission from Naturalis Biodiversity Center http://www.repository.naturalis.nl/record/261776). **Abbreviations**: **art**, angulo-articular; **BL**, body length; **h,** minimal body height; **dent**, dentary; **H**, maximal body height; **H2**, body height at origin of anal fin; **HD**, head depth; **HL**, Head length; **lA**, length of anal fin base; **lasc**, length of premaxillary ascending process; **lC**, length of caudal fin; **lD**, length of dorsal fin base; **lLj**, length of lower jaw; **lpc**, length of caudal peduncle; **lpmx**, length of premaxilla; **lV**, length of pelvic fin base; **lVsp**, length of pelvic fin spine; **pD**, postdorsal distance; **pmx**, premaxilla; **prA**, preanal distance; **prD**, predorsal distance; **prV**, prepelvic distance; **rart**, retro-articular; **SL**, standard length; **TL**, total length.

**Additional file 4.** Supplementary Data S4. Text file used for the phylogenetic analysis on the basis of the matrix of Stiassny [85].

**Additional file 5.** Character list and states used for phylogenetic analysis (compiled from Stiassny, [85]).

**Additional file 6 **Supplementary Fig. S2. Principal coordinates analysis (PCoA) scatter plot. Analysis based on eight meristic characters and the number of supraneurals from the 16 pseudocrenilabrine tribes (26 lineages) shown in Fig. 7 (*N* = 854; see Additional file 2: Supplementary Data S2 for raw data) and from †*Warilochromis unicuspidatus* gen. et sp. nov. Species score limits are visualized as 95% confidence ellipses. Note that the ellipses shown encompass only members of the tribes that overlap with †*Warilochromis*. Coordinate 1 explains 47.13% and Coordinate 2 explains 40.75% of the variation.

## Data Availability

The datasets used and analysed during the current study are available in Additional file 1: Supplementary Data S1, Additional file 2: Supplementary Data S2 and Additional file 4: Supplementary Data S4.

## Abbreviations

#LL: number of lateral-line segments on the body
#Lt: number of lateral-line tubules on the lacrimal
#Sn: number of supraneural bones
ach: anterior ceratohyal
art: angulo-articular
bh: basihyal
bp & bp´: basipterygium
cl & cl´: cleithrum
co: coracoid
dent: dentary
dhh: dorsal hypohyal
e: ectopterygoid
ep1, ep2: epurals
Fm: Formation
HPD: Highest posterior intervals
hs: haemal spines
hy1–5: hypural plates
hyo & hyo´: hyomandibula
Hyp 1–2, 3–4: configuration of the hypural plates
io1–4: infraorbitals 1–4
lac & lac´: lacrimal
LTR: Lake Tanganyika Radiation
m.y.: million years
MPT: Most parsimonious tree
mx: maxilla
MYA: Million years ago
ns: neural spines
o: otolith imprint
op: opercle
pa: palatine
pch: posterior ceratohyal
PCoA: Principal coordinates analysis
ph: parhypural
pmx & pmx´: premaxilla
pop: preopercle
psp: parasphenoid
ptt & ptt´: posttemporal
pu: preural centrum
s: symplectic
scl: supracleithrum
SD: Standard deviation
SL: Standard length
sn: supraneural
SNSB – BSPG: Bavarian State Collection of Palaeontology and Geology, Munich, Germany
SNSB – ZSM: Bavarian State Collection of Zoology, Munich, Germany
sop: suboperculum
un: uroneural
us: terminal centrum
v: vomer
vhh & vhh´: ventral hypohyal
Vt: total number of vertebrae
VtPtLDs: ordinal number (s) of vertebrae associated with the pterygiophore of the last dorsal fin spine
+: present
−: absent
