# Supplementary material for: New haplochromine cichlid from the upper Miocene (9–10 MYA) of Central Kenya
Source: BMC Evol Biol. 2020 Jun 5;20:65. doi: 10.1186/s12862-020-01602-x (PMC7275555; doi:10.1186/s12862-020-01602-x)
Supplement: Supplementary file 1 — Additional file 1. Supplementary Data S1. Comparative material of extant species used for X-ray analysis and assembly of meristic data. [file 12862_2020_1602_MOESM1_ESM.doc]

**Supplementary Data S1. Comparative material of extant species used for X-ray analysis and assembly of meristic data.**

**Institutional abbreviations**: **BMNH**, Natural History Museum, London, UK; **CU**, Cornell University Museum of Vertebrates, Ithaca, New York, USA; **IRSNB**, Institut Royal des Sciences Naturelles de Belgique, Brussels, Belgium; **MRAC**, Musée Royal de l´Afrique Centrale, Tervuren, Belgium; **ZSM**, Bavarian State Collection of Zoology, Munich, Germany.

Subfamily PSEUDOCRENILABRINAE, **Hemichromines**: *Hemichromis bimaculatus* Gill, 1862 (ZSM 035696_1, ZSM 035696_2), *Hemichromis cerasogaster* (Boulenger, 1899)

(ZSM 042327), *Hemichromis* cf. *fasciatus* (ZSM 036150), *Hemichromis cristatus* Loiselle, 1979 (ZSM 040942), *Hemichromis elongatus* (Guichenot, 1861) (ZSM 039982_1, ZSM 039982_2), *Hemichromis fasciatus* Peters, 1857 (ZSM 036143, ZSM 036144), *Hemichromis guttatus* Günther, 1862 (ZSM 043121), *Hemichromis letourneuxi* Sauvage, 1880 (ZSM 040946_(P-AA-0617), ZSM 040946_(P-AA-0619)), *Hemichromis lifalili* Loiselle, 1979 (ZSM 039385, ZSM 039491), *Hemichromis* sp. "Ankasa" (ZSM 039633_(P-AA-0389), ZSM 039633_(P-AA-0390)), *Hemichromis* sp. "Bioko" (ZSM 027617_2, ZSM 027617_3), *Hemichromis stellifer* Loiselle, 1979 (ZSM 038398_(DRC-2008+0509), ZSM 038398_(DRC-2008+0511)).

**Pelmatochromines**: *Pelmatochromis nigrofasciatus* (Pellegrin, 1900) (ZSM 029650_1, ZSM 029650_2), *Pelmatochromis* sp. (IRSNB A-0628), *Pelmatochromis* sp. (ZSM 041808_1, ZSM 041808_2), *Pterochromis congicus* (Boulenger, 1897) (ZSM 029697, ZSM 029701).

**Chromidotilapiines**: *Benitochromis* cf. *batesii* (ZSM 024904), *Benitochromis* cf. *batesii* "Akok II" (ZSM 043096_(P-AA-0964), ZSM 043096_(P-AA-0973)), *Benitochromis* cf. *batesii* "Akok" (ZSM 043095_(P-AA-0971), ZSM 043095_(P-AA-0972)), *Benitochromis* cf. *batesii* "Makondo" (ZSM 043090), *Benitochromis* cf. *batesii* "Streched" (ZSM 043092_(PAA-0967), ZSM 043092_1), *Benitochromis finleyi* (Trewavas, 1974) (ZSM 043094), *Benitochromis* indet. (ZSM 024916_2, ZSM 025151_1, ZSM 025151_2, ZSM 025151), *Benitochromis riomuniensis* (Thys van den Audenaerde, 1981) (ZSM 043091), *Benitochromis* sp. (ZSM 036643), *Benitochromis ufermanni* Lamboj, 2001 (ZSM 043093_(P-AA-0968), ZSM 043093_(P-AA-0969)), *Chromidotilapia cavalliensis* (Thys van den Audenaerde and Loiselle, 1971) (ZSM 027410), *Chromidotilapia guntheri bosumtwensis* (ZSM 036131_(GHA-2002+187),ZSM 036131_(GHA-2002+188)), *Chromidotilapia guntheri guntheri* (ZSM 039977_1, ZSM 039977_2, ZSM 027425, ZSM 035989_1, ZSM 035989_2, ZSM 035991_1, ZSM 035991_2, ZSM 039886_1, ZSM 039886_2, ZSM 039955, ZSM 039958, ZSM 043011_(SUD-2014-450), ZSM 043011_(SUD-2014-451), ZSM 043011_(SUD-2014-463)), *Chromidotilapia guntheri loennbergi* (ZSM 036568_1, ZSM 036568_2), *Chromidotilapia schoutedeni* (Poll and Thys van den Audenaerde, 1967) (IRSNB A_0691_1, IRSNB A_0691_2, ZSM 037559), *Chromidotilapia* sp. (IRSNB A-0662_1,

IRSNB A-0662_2), *Congochromis dimidiatus* (Pellegrin, 1900) (ZSM 025027_1, ZSM 025027_2), *Congochromis sabinae* (Lamboj, 2005) (ZSM 039478_0776, ZSM 039478_0777), *Divandu albimarginatus* Lamboj and Snoeks, 2000 (ZSM uncat_1_Divandu, ZSM uncat_2_Divandu), *Limbochromis robertsi* (Thys van den Audenaerde and Loiselle, 1971) (ZSM 09726_1, ZSM 09726_2), *Nanochromis nudiceps* (Boulenger, 1899) (ZSM 037364_1, ZSM 037364_2), *Nanochromis wickleri* Schliewen and Stiassny, 2006 (ZSM 029696, ZSM 033989a_1), *Parananochromis axelrodi* Lamboj and Stiassny, 2003 (ZSM 042312), *Parananochromis caudifasciatus* (Boulenger, 1913) (ZSM 034253_1, ZSM 034253_2), *Parananochromis longirostris* (Boulenger ,1903) (ZSM uncat_1_Parananochromis, ZSM uncat_2_Parananochromis), *Pelvicachromis* (*Enigmatochromis*) *lucanusi* Lamboj, 2009 (ZSM 039579), *Wallaceochromis* (*Pelvicachromis*) *humilis* (Boulenger, 1916) (ZSM 023718), *Pelvicachromis taeniatus* (Boulenger, 1901) (ZSM 035995_1), *Teleogramma brichardi* Poll, 1959 (ZSM 019076_1, ZSM 019076_2), *Teleogramma gracile* Boulenger, 1899 (ZSM 038127_(DRC-2008+0314), ZSM 038127_(DRC-2008+0321)), Teleogramma sp. (ZSM 038386_(DRC-2008+0201), ZSM 038386_(DRC-2008+0213)).

Of the two genera and four species of the Pelmatochromines our new dataset includes all, but not *Pelmatochromis buettikoferi* and *P. ocellifer*. An almost complete data set is also available for the Hemichromines (two genera, 12 species) with exception of *Anomalochromis* and *Hemichromis angolensis*, *H. exsul*, *H. fasciatus*, and *H. paynei*. The Chromidotilapiines comprise a total of 12 genera and 58 species. Of these, our dataset does not include *Benitochromis conjunctus*, *B. nigrodorsalis*, *Chromidotilapia elongata*, *C. kingsleyae*, *C. linkei*, *C. mamonekenei*, *C. melaniae*, *C. mrac*, *C. nana*, *C. regani*, *Congochromis pugnatus*, *C. robustus*, *C. squamiceps*, *Nanochromis consortus*, *N. minor*, *N. parilus*, *N. splendens*, *N. teugelsi*, *N. transvestitus*, *Parananochromis brevirostris*, *P. elobatus*, *P. gabonicus*, *P. ornatus*, *P. orsorum*, *Pelvicachromis drachenfelsi*, *P. kribensis*, *P. pulcher*, *P. roloffi*, *P. sacrimontis*, *P. silviae*, *P. subocellatus*, *Teleogramma depressa*, *T. monogramma*, *T. obamaorum*, *Thysochromis ansorgii*, *Walaceochromis rubrolabiatus*, and *W. signatus*.

Tribe **Boulengerochromini**: *Boulengerochromis microlepis* (Boulenger, 1899) (ZSM 040843, MRAC 74-06-P-18-21_1, MRAC 74-06-P-18-21_2, MRAC 74-06-P-18-21_3, MRAC 74-06-P-18-21_4, MRAC -107323-107328_1, MRAC -107323-107328_2, MRAC -107323-107328_3, MRAC -107323-107328_4).

Tribe **Bathybatini**: *Bathybates fasciatus* Boulenger, 1901 (ZSM 041479_(DRC-2011+0949), ZSM 041479_(DRC-2011+0950), MRAC -112222-112225_1, MRAC -112222-112225_2, MRAC -112222-112225_3), *Bathybates ferox* Boulenger, 1898 (MRAC -112181-112186_1, MRAC -112181-112186_2, MRAC -112181-112186_3, MRAC -112181-112186_4, MRAC -112181-112186_5, MRAC -112181-112186_6), *Bathybates graueri* Steindachner, 1911 (MRAC -112463-112467_1, MRAC -112463-112467_2, MRAC -112463-112467_3, MRAC -112463-112467_4, MRAC -112463-112467_5), *Bathybates hornii* Steindachner, 1911 (MRAC -P112482-112483_1, MRAC -P112482-112483_2), *Bathybates leo* Poll, 1956 (MRAC -112492-112496_1, MRAC -112492-112496_2, MRAC -112492-112496_3, MRAC -112492-112496_4), *Bathybates minor* Boulenger, 1906 (MRAC -94-069-P-1025-1028_1, MRAC -94-069-P-1025-1028_2, MRAC -94-069-P-1025-1028_3, MRAC -94-069-P-1025-1028_4, MRAC -96-031-P-1238-1271_1, MRAC -96-031-P-1238-1271_2, MRAC -96-031-P-1238-1271_3, MRAC -96-031-P-1238-1271_4, MRAC -96-031-P-1238-1271_5, MRAC -96-031-P-1238-1271_6, MRAC -96-031-P-1238-1271_7, MRAC -96-031-P-1238-1271_8, MRAC -96-031-P-1238-1271_9, MRAC -96-031-P-1238-1271_10, MRAC-96-031-P-1238-1271_11, MRAC -96-031-P-1238-1271_12, MRAC -96-031-P-1238-1271_13, MRAC -96-031-P-1238-1271_14, MRAC -96-031-P-1238-1271_15, MRAC -96-031-P-1238-1271_16, MRAC -96-031-P-1238-1271_17, MRAC -96-031-P-1238-1271_18), *Bathybates vittatus* Boulenger, 1914 (MRAC -P-112487-112488_1, MRAC -P-112487-112488_2), *Hemibates stenosoma* (Boulenger, 1901) (MRAC -94-069-P-1032-1034_1, MRAC -94-069-P-1032-1034_2, MRAC -94-069-P-1032-1034_3, MRAC -P-112134-112135_1, MRAC -P-112134-112135_2).

Tribe **Trematocarini**: *Trematocara kufferathi* Poll, 1948 (MRAC -94-069-P-1048-1096_1, MRAC -94-069-P-1048-1096_2, MRAC -94-069-P-1048-1096_3, MRAC -94-069-P-1048-1096_4, MRAC -94-069-P-1048-1096_5, MRAC -94-069-P-1048-1096_6, MRAC -94-069-P-1048-1096_7, MRAC -94-069-P-1048-1096_8, MRAC -94-069-P-1048-1096_9, MRAC -94-069-P-1048-1096_10, MRAC -94-069-P-1048-1096_11, MRAC -94-069-P-1048-1096_12, MRAC -94-069-P-1048-1096_13, MRAC -94-069-P-1048-1096_14, MRAC -94-069-P-1048-1096_15, MRAC -94-069-P-1048-1096_16, MRAC -94-069-P-1048-1096_17, MRAC -94-069-P-1048-1096_18), *Trematocara* *macrostoma* Poll, 1952 (MRAC -B0-012-P-0526-0530_1, MRAC -B0-012-P-0526-0530_4), *Trematocara marginatum* Boulenger, 1899 (ZSM 039570, MRAC -94-069-P-1739-1758_1, MRAC -94-069-P-1739-1758_2, MRAC -94-069-P-1739-1758_3, MRAC -94-069-P-1739-1758_4, MRAC -94-069-P-1739-1758_5, MRAC -94-069-P-1739-1758_7, MRAC -94-069-P-1739-1758_8, MRAC -94-069-P-1739-1758_9, MRAC-94-069-P-1739-1758_10, MRAC -94-069-P-1739-1758_11, MRAC -94-069-P-1739-1758_12, MRAC -94-069-P-1739-1758_13, MRAC -94-069-P-1739-1758_14, MRAC -94-069-P-1739-1758_15, MRAC -94-069-P-1739-1758_16, MRAC -94-069-P-1739-1758_17, MRAC -94-069-P-1739-1758_19), *Trematocara nigrifrons* Boulenger, 1906 (MRAC -94-069-P-0392-0417_1, MRAC -94-069-P-0392-0417_2, MRAC -94-069-P-0392-0417_3, MRAC -94-069-P-0392-0417_4, MRAC -94-069-P-0392-0417_5, MRAC -94-069-P-0392-0417_6, MRAC -94-069-P-0392-0417_7, MRAC -94-069-P-0392-0417_8, MRAC -94-069-P-0392-0417_12, MRAC -94-069-P-0392-0417_15), *Trematocara stigmaticum* Poll, 1943 (MRAC -95-089-P-0298-0322_1, MRAC -95-089-P-0298-0322_2, MRAC -95-089-P-0298-0322_3, MRAC -95-089-P-0298-0322_4, MRAC -95-089-P-0298-0322_5, MRAC -95-089-P-0298-0322_6, MRAC -95-089-P-0298-0322_7, MRAC -95-089-P-0298-0322_8, MRAC -95-089-P-0298-0322_9, MRAC -95-089-P-0298-0322_12, MRAC -95-089-P-0298-0322_13, MRAC -95-089-P-0298-0322_17, MRAC -95-089-P-0298-0322_18), *Trematocara unimaculatum* Boulenger, 1901 (MRAC -94-069-P-1362-1374_1, MRAC -94-069-P-1362-1374_2, MRAC -94-069-P-1362-1374_3, MRAC -94-069-P-1362-1374_4, MRAC -94-069-P-1362-1374_5, MRAC -94-069-P-1362-1374_6, MRAC -94-069-P-1362-1374_7, MRAC -94-069-P-1362-1374_8, MRAC -94-069-P-1362-1374_9, MRAC -94-069-P-1362-1374_10, MRAC -94-069-P-1362-1374_11, MRAC -94-069-P-1362-1374_12, MRAC -94-069-P-1362-1374_13), *Trematocara variabile* Poll, 1952 (MRAC -94-069-P-1810-1891_1, MRAC -94-069-P-1810-1891_2, MRAC -94-069-P-1810-1891_3, MRAC -94-069-P-1810-1891_4, MRAC -94-069-P-1810-1891_5, MRAC -94-069-P-1810-1891_6, MRAC -94-069-P-1810-1891_7, MRAC -94-069-P-1810-1891_8, MRAC -94-069-P-1810-1891_9, MRAC -94-069-P-1810-1891_16), *Trematocara* cf. *variabile* Poll, 1952 (ZSM 042334), *Trematocara zebra* De Vos, Nshombo and Thys van den Audenaerde, 1996 (MRAC -96-083-P-0760-0762_1, MRAC -96-083-P-0760-0762_2, MRAC -96-083-P-0760-0762_3).

Tribe **Benthochromini**: *Benthochromis horii* Takahashi, 2008 (MRAC -A7-020-P-0001-0003_1, MRAC -A7-020-P-0001-0003_2, MRAC -A7-020-P-0001-0003_3), *Benthochromis melanoides* (Poll, 1984) (MRAC -112548), *Benthochromis tricoti* (Poll, 1948) (ZSM 040833, ZSM 043239_(P-AA-0997), ZSM 043239_(P-AA-0998), MRAC -94-069-P-1573-1578_1, MRAC -94-069-P-1573-1578_2, MRAC -94-069-P-1573-1578_3, MRAC -94-069-P-1573-1578_4, MRAC -94-069-P-1573-1578_5, MRAC -94-069-P-1573-1578_6).

Tribe **Cyprichromini**: *Cyprichromis leptosoma* “Kigoma” (Boulenger, 1898) (ZSM 043238_(P-AA-0995), ZSM 043238-(P-AA-0996)), *Cyprichromis microlepidotus* (Poll, 1956) (ZSM 040807), *Cyprichromis zonatus* Takahashi, Hori and Nakaya, 2002 (ZSM 040812), *Cyprichromis zonatus* “Chituta” Takahashi, Hori and Nakaya, 2002 (ZSM 042935), *Paracyprichromis nigripinnis* (Boulenger, 1091) (ZSM 040793).

Tribe **Perissodini**: *Haplotaxodon microlepis* Boulenger, 1906 (ZSM 040840, ZSM 040839, ZSM 043243_(P-AA-1005), ZSM 043243_(P-AA-1006)), *Perissodus microlepis* Boulenger, 1898 (ZSM 042332_(P-AA-0582), ZSM 042331_(P-AA-0583)), *Plecodus multidentatus* Poll, 1952 (MRAC -95-098-P-0266-0267_1, MRAC -95-098-P-0266-0267_2), *Xenochromis hecqui* Boulenger, 1899 (MRAC -112569-112572-1, MRAC -112569-112572-2).

Tribe **Cyphotilapiini**: *Cyphotilapia* sp. (ZSM 043240_(P-AA-0999), ZSM 043240_(P-AA-1000), ZSM 040818, ZSM 040815), *Trematochromis benthicola* (Matthes, 1962) (MRAC -96-083-P-0764-0772_1, MRAC -96-083-P-0764-0772_2, MRAC -96-083-P-0764-0772_3, MRAC -96-083-P-0764-0772_4, MRAC -96-083-P-0764-0772_5, MRAC -96-083-P-0764-0772_6, MRAC -96-083-P-0764-0772_7, MRAC -96-083-P-0764-0772_8, MRAC -96-083-P-0764-0772_9, ZSM 043236).

Tribe **Limnochromini**: *Gnathochromis permaxillaris* (David, 1936) (ZSM 040819), *Greenwoodochromis bellcrossi* (Poll, 1976) (ZSM 042335), *Limnochromis auritus* (Boulenger, 1901) (ZSM 043242_(P-AA-1003), ZSM 043242_(P-AA-1004)), *Limnochromis staneri* Poll, 1949 (ZSM 040846), *Reganochromis calliurus* (Boulenger, 1901) (ZSM 040799), *Tangachromis* *dhanisi* (Poll, 1949) (MRAC -107296-107300_1, MRAC -107296-107300_2, MRAC -107296-107300_3, MRAC -107296-107300_4), *Triglachromis* *otostigma* (Regan, 1920) (ZSM 024837_1, ZSM 024837_2, ZSM 040847).

Tribe **Ectodini**: *Asprotilapia leptura* Boulenger, 1901 (MRAC -92-081-P-2250-2253_1, MRAC -92-081-P-2250-2253_2, MRAC -92-081-P-2250-2253_3, MRAC -92-081-P-2250-2253_4), *Aulonocranus dewindti* (Boulenger, 1899) (MRAC -81-062-P-0066-0074_1, MRAC -81-062-P-0066-0074_2, MRAC -81-062-P-0066-0074_3, MRAC -81-062-P-0066-0074_4, MRAC -81-062-P-0066-0074_5, MRAC -81-062-P-0066-0074_6, MRAC -81-062-P-0066-0074_7, MRAC -81-062-P-0066-0074_8, MRAC -81-062-P-0066-0074_9), *Callochromis macrops* (Boulenger, 1898) (ZSM 040823), *Callochromis melanostigma* (Boulenger, 1906) (ZSM 042931, ZSM 024765), *Callochromis pleurospilus* (Boulenger, 1906) (ZSM 024764), *Callochromis* *stappersii* (Boulenger, 1914) (ZSM 040870), *Cardiopharynx schoutedeni* Poll, 1942 (MRAC -94-069-P-1581-1591_1, MRAC -94-069-P-1581-1591_2, MRAC -94-069-P-1581-1591_3, MRAC -94-069-P-1581-1591_4, MRAC -94-069-P-1581-1591_5), *Cunningtonia longiventralis* Boulenger, 1906 (ZSM 040848), *Cyathopharynx furcifer* (Boulenger, 1898) (ZSM 040811), *Ectodus descampsi* Boulenger, 1898 (ZSM 040810), *Grammatotria lemairii* Boulenger, 1899 (ZSM 040826), *Lestradea perspicax* Poll, 1943 (MRAC -P42674-42677_1, MRAC -P42674-42677_2, MRAC -P42674-42677_3, MRAC -P42674-42677_4), *Ophthalmotilapia ventralis* (Boulenger, 1898) (ZSM 043241-(P-AA-1001), ZSM 043241_(P-AA-1002)), *Xenotilapia flavipinnis* Poll, 1985 (ZSM 040836), *Xenotilapia melanogenys* (Boulenger, 1898) (ZSM 040813), *Xenotilapia ornatipinnis* Boulenger, 1901 (ZSM 040834), *Xenotilapia rotundiventralis* (Takahashi, Yanagisawa and Nakaya 1997) (ZSM 042934), *Xenotilapia sima* Boulenger, 1899 (BMNH 1961.11.22.222-224_1, BMNH 1961.11.22.222-224_2, BMNH 1994.11.3.65-68_1, BMNH 1994.11.3.65-68_2, BMNH 1899.11.27.103_1 Syntype, BMNH 1899.11.27.103_2 Syntype), *Xenotilapia spiloptera* Poll and Stewart, 1975 (ZSM 040838).

Tribe **Lamprologini**: *Altolamprologus calvus* (Poll, 1978) (ZSM 040877), *Altolamprologus, compressiceps* (Boulenger, 1898) (ZSM 040871), *Chalinochromis brichardi* Poll, 1974 (ZSM 040879), *Chalinochromis popelini* Brichard 1989 (ZSM 040820), *Chalinochromis* sp. “Ndobhoi” (ZSM 040878), *Julidochromis marlieri* Poll, 1956 (ZSM 040805), *Julidochromis ornatus* Boulenger, 1898 (ZSM 040788), *Julidochromis regani* Poll, 1942 (ZSM 040806), *Lamprologus lemairii* Boulenger, 1899 (ZSM 024240_1, ZSM 024240_2), *Lamprologus mocquardi* Pellegrin, 1903 (ZSM 038370_1, ZSM 038370_2), *Lamprologus* sp. (ZSM 038102_(DRC-2008+435), ZSM 038102_(DRC-2008+437)), *Lamprologus speciosus* Büscher, 1991 (ZSM 027972_1, ZSM 027972_2), *Lamprologus teugelsi* Schelly and Stiassny, 2004 (ZSM 038141_(DRC-2008+150), ZSM 038141_(DRC-2008+151)), *Lepidiolamprologus* *cunningtoni* (Boulenger, 1906) (ZSM 024336), *Lepidiolamprologus elongatus* (Boulenger, 1898) (ZSM 024239_1, ZSM 024239_2, ZSM 024239_4), *Lepidiolamprologus* *nkambae* (Staeck, 1978) (ZSM 040795), *Neolamprologus* *brichardi* (Poll, 1974) (ZSM 033918_1, ZSM 033918_2, ZSM 040802), *Neolamprologus calliurus* (Boulenger, 1906) (ZSM 040824), *Neolamprologus nigriventris* Büscher 1992 (ZSM 028413_3, ZSM 028413_4), *Neolamprologus pectoralis* Büscher, 1991 (ZSM 028095), *Neolamprologus prochilus* (Bailey and Stewart, 1977) (ZSM 040924, *Neolamprologus similis* Büscher, 1992 (ZSM 028383), *Neolamprologus* sp. (ZSM 040800), *Neolamprologus toae* (Poll, 1949) (MRAC -94-069-P-0779-0787_1, MRAC -94-069-P-0779-0787_2, MRAC -94-069-P-0779-0787_3, MRAC -94-069-P-0779-0787_4, MRAC -94-069-P-0779-0787_5, MRAC -94-069-P-0779-0787_6, MRAC -94-069-P-0779-0787_7, MRAC -94-069-P-0779-0787_9), *Telmatochromis dhonti* (Boulenger, 1919) (ZSM 024200_1), *Telmatochromis* sp. (ZSM 040829, *Telmatochromis temporalis* Boulenger, 1898 (ZSM 024984, ZSM 024237_1, ZSM 024237_2), *Variabilichromis moorii* (Boulenger, 1898) (ZSM 040832).

Tribe **Eretmodini**: *Eretmodus cyanostictus* Boulenger, 1898 (ZSM 040841, ZSM 024172_1, ZSM 024172_2, ZSM 024175_1, ZSM 024175_2, ZSM 024175_3, ZSM 043237_(P-AA-0993), ZSM 043237_(P-AA-0994)), *Spathodus erythrodon* Boulenger, 1900 (ZSM 040844), *Spathodus marlieri* Poll, 1950 (ZSM 040842), *Tanganicodus irsacae* Poll, 1950 (ZSM 042333).

Tribe **Haplochromini (Tropheini)**: *Gnathochromis pfefferi* (Boulenger, 1898) (ZSM 024242, ZSM 040923), *Haplochromis horei* (Günther, 1894) (ZSM 024846_1), *Limnotilapia dardennii* (Boulenger, 1899) (ZSM 024944_1, ZSM 040925), *Lobochilotes labiatus* (Boulenger, 1898) (ZSM 040922, ZSM 024174_1), *Petrochromis trewavasae* Poll, 1948 (ZSM 040831), *Simochromis babaulti* Pellegrin, 1927 (ZSM 040927), *Simochromis diagramma* (Günther, 1894) (ZSM 040926), *Tropheus duboisi* Marlier, 1959 (ZSM 040792), *Tropheus moorii* Boulenger, 1898 (ZSM 024982_1, ZSM 024982_2, ZSM 024982_3, ZSM 024339_1, ZSM 024339_2, ZSM 024339_3, ZSM 040828), *Tropheus polli* Axelrod, 1977 (ZSM 040790).

Tribe **Haplochromini (*Haplochromis vanheusdeni*):** *Haplochromis vanheusdeni* Schedel, Friel and Schliewen, 2014 (ZSM 042320, ZSM 042311, ZSM 041559_1, ZSM 041559_2, ZSM 041559_3, ZSM 041559_4, ZSM 041559_5, ZSM 041559_6, ZSM 041559_7, ZSM 041559_8, ZSM 041559_9, ZSM 041559_10, ZSM 041559_11, ZSM 041440_1, ZSM 041440_2, ZSM 041440_3, ZSM 043134).

**Species of *Orthochromis*** (Malagarasi-*Orthochromis*): *Orthochromis kasuluensis* De Vos and Seegers, 1998 (ZSM 041455_1, ZSM 041455_2, ZSM 041455_3, ZSM 041455_4, ZSM 041455_5), *Orthochromis luichensis* De Vos and Seegers, 1998 (ZSM 041445, ZSM 041445_2, ZSM 041445_3, ZSM 041445_4, ZSM 041445, ZSM 041445_6, ZSM 041445_7), *Orthochromis malagaraziensis* (David, 1937) (ZSM 41469_(DRC-2001+1029), ZSM 41469_(DRC-2001+1030)), *Orthochromis rubrolabialis* De Vos and Seegers, 1998 (ZSM 041463_1, ZSM 041463_2, ZSM 041463_3, ZSM 041463_4, ZSM 041463_5, ZSM 041463_6, ZSM 041463_7, ZSM 041463_8), *Orthochromis* sp. Igamba (ZSM 041563_(P-AA-1077), ZSM 041563_(P-AA-1078), ZSM 041563_(P-AA-1079), ZSM 041561_1, ZSM 041561_2, ZSM 041561_3, ZSM 041561_4, ZSM 041561_5), *Orthochromis uvinzae* De Vos and Seegers, 1998 (ZSM 041564_1, ZSM 041564_2, ZSM 041564_3, ZSM 041564_4, ZSM 041564_5, ZSM 041562_1, ZSM 041562_2, ZSM 041562_3, ZSM 041562_4, ZSM 041562_5, ZSM 041430_1, ZSM 041430_2, ZSM 041430_3, ZSM 041430_4, ZSM 041430_5, ZSM 041430_6, ZSM 041430_7).

**Species of *Orthochromis*** (Northern-Zambian-*Orthochromis*): *Orthochromis kalungwishiensis* (Greenwood and Kullander, 1994) (ZSM 041431_1, ZSM 041431_2, ZSM 041431_3, ZSM 041431_4, ZSM 041431_5, ZSM 041431_7, ZSM 041427), *Orthochromis luongoensis* (Greenwood and Kullander, 1994) (CU 91747, ZSM 041437_1, ZSM 041437_2, ZSM 041437_3, ZSM 041437_4, ZSM 041437_5, ZSM 041437_6, ZSM 044432_6998), *Orthochromis* sp. Kashinsa (ZSM 041443_1, ZSM 041443_2, ZSM 041443_3, ZSM 041443_4, ZSM 041443_5, ZSM 041429_1, ZSM 041429_2, ZSM 041429_3, ZSM 041429_4, ZSM 041429_5, ZSM 041429_6, ZSM 041429_7, ZSM 041429_8, ZSM 041429_9, ZSM 041429_10, ZSM 041429_11), *Orthochromis* sp. Mambilima (ZSM 042322_(P-AA-0698), ZSM 042322_(P-AA-0701), ZSM 041450_1, ZSM 041450_2, ZSM 041450_3, ZSM 041450_4, ZSM 041450_5, ZSM 041450_6, ZSM 041450_7, ZSM uncatalogued specimen).

**Species of *Orthochromis*** (LML-*Orthochromis*): *Orthochromis stormsi* (Boulenger, 1902) (ZSM 032359_1, ZSM 032359_2 ZSM 042319, ZSM 032410_1, ZSM 032410_2, ZSM 023693, ZSM 032393_1, ZSM 032393_2, ZSM 032393_3, ZSM 032393_4, ZSM 032393_5, ZSM 037541_1, ZSM 037541_2, ZSM 037541_3, ZSM 037603, ZSM 038337, ZSM 038129_1, ZSM 038129_2, ZSM 038129_3, ZSM 042319, ZSM 042318).

**Species of *Orthochromis*** (*Orthochromis machadoi*): *Orthochromis machadoi* (Poll, 1967) (BMNH 1984.2.6.104-108_1, BMNH 1984.2.6.104-108_2, BMNH 1984.2.6.104-108_3, BMNH 1984.2.6.104-108_4, BMNH 1984.2.6.104-108_5, BMNH 1984.2.6.109, BMNH 1984.2.6.113, BMNH 1984.2.6.116-131_2, BMNH 1984.2.6.116-131_3, BMNH 1984.2.6.116-131_4, BMNH 1984.2.6.132-141_1, BMNH 1984.2.6.132-141_2, BMNH 1984.2.6.132-141_3, BMNH 1984.2.6.142-145_1, BMNH 1984.2.6.142-145_2, BMNH 1984.2.6.142-145_3, BMNH 1984.2.6.142-145_4).

**Species of *Orthochromis*** (*Orthochromis torrenticola*): *Orthochromis torrenticola* (Thys van den Audenaerde, 1963) (ZSM 038201_(Uli-LUB-2008+008), ZSM 038201_2, ZSM 038201_4, ZSM 038201_5).

Tribe **Haplochromini (Lake Malawi Haplochromini):** *Haplochromis callipterus* (Günther, 1894) (ZSM 040907, *Haplochromis latifasciatus* Regan, 1929 (ZSM 040865), *Abactochromis labrosus* (Trewavas, 1935) (ZSM 040852), *Alticorpus macrcoleithrum* (Stauffer and McKaye, 1985) (MRAC-99-041-P-3713-3715_1, MRAC-99-041-P-3713-3715_2, MRAC-99-041-P-3713-3715_3), *Alticorpus mentale* Stauffer and McKaye, 1988 (MRAC-99-041-P-3685-3687_1, MRAC-99-041-P-3685-3687_2, MRAC-99-041-P-3685-3687_3), *Aristochromis christyi* Trewavas, 1935 (ZSM 040967_5461), *Aulonocara aquilonium* Konings, 1995 (ZSM 040889), *Aulonocara baenschi* Meyer and Riehl, 1985 (ZSM 040883), *Aulonocara nyassae* Regan, 1922 (ZSM 041198_5436, ZSM 041198_5439), *Aulonocara rostratum* Trewavas, 1935 (ZSM 040893), *Aulonocara saulosi* Meyer, Riehl and Zetzsche, 1987 (ZSM 041170_5143, ZSM 041170_5145, ZSM 041170_5146), *Aulonocara steveni* Meyer, Riehl and Zetzsche, 1987 (ZSM 041162-5039, ZSM 041162_5040),

*Buccochromis heterotaenia* (Trewavas, 1935) (ZSM 040894)*, Caprichromis* ?*liemi* (McKaye and MacKenzie, 1982) (ZSM 041179_5652, ZSM 041179_5653, ZSM 041179_5654), *Champsochromis spilorhynchus* (Regan, 1922) (ZSM 040909, ZSM 040909_1), *Cheilochromis euchilus* (Trewavas, 1935) (ZSM 040905), *Copadichromis borleyi* (Iles, 1960) (ZSM 041190_5335, ZSM 041190_5336), *Corematodus* indet. (ZSM 041195_5459), *Corematodus* sp. (ZSM 041219_5325), *Ctenopharynx nitidus* (Trewavas, 1935) (ZSM 040895), *Cyathochromis obliquidens* Trewavas, 1935 (MRAC-99-041-P-1790-1804_1, MRAC-99-041-P-1790-1804_2, MRAC-99-041-P-1790-1804_3, MRAC-99-041-P-1790-1804_4, MRAC-99-041-P-1790-1804_5, MRAC-99-041-P-1790-1804_6, MRAC-99-041-P-1790-1804_7, MRAC-99-041-P-1790-1804_8, MRAC-99-041-P-1790-1804_9), *Cynotilapia afra* (Günther, 1894) (ZSM 041217_5014, ZSM 041217_5016, ZSM 041217_5020), *Dimidiochromis compressiceps* (Boulenger, 1908) (ZSM 024969), *Dimidiochromis kiwinge* (Ahl 1926) (ZSM 041169_5316), *Diplotaxodon apogon* Turner and Stauffer, 1998 (MRAC-99-041-P-10750-10753-1, MRAC-99-041-P-10750-10753-2, MRAC-99-041-P-10750-10753-3, MRAC-99-041-P-10750-10753-4), *Diplotaxodon limnothrissa* Turner, 1994 (MRAC-99-041-P-5341-5345_1, MRAC-99-041-P-5341-5345_2, MRAC-99-041-P-5341-5345_3, MRAC-99-041-P-5341-5345_4, MRAC-99-041-P-5341-5345_5), *Diplotaxodon macrops* Turner and Stauffer, 1998 (MRAC-99-041-P-10763), *Diplotaxodon* sp. (MRAC-99-041-P-10740-10742_2, MRAC-99-041-P-10740-10742_3), *Eclectochromis lobochilus* (Trewavas, 1935) (ZSM 040886), *Exochochromis anagenys* Oliver, 1989 (ZSM 040887), *Fossorochromis rostratus* (Boulenger, 1899) (ZSM 040882), *Genyochromis mento* Trewavas, 1935 (MRAC-99-041-P-1993-1996_1, MRAC-99-041-P-1993-1996_2, MRAC-99-041-P-1993-1996_3, MRAC-99-041-P-1993-1996_4, ZSM 027946), *Hemitaeniochromis urotaenia* (Regan, 1922) (MRAC-99-041-P-1741-1745_1, MRAC-99-041-P-1741-1745_2, MRAC-99-041-P-1741-1745_3, MRAC-99-041-P-1741-1745_4, MRAC-99-041-P-1741-1745_5), *Hemitilapia oxyrhyncha* Boulenger, 1902 (MRAC-99-041-P-1767-1768_1, MRAC-99-041-P-1767-1768_2), *Labeotropheus trewavasae* Fryer, 1956 (ZSM 040853), *Labidochromis chisumulae* Lewis, 1982 (ZSM 040851), *Labidochromis* sp. “yellow” (ZSM 040007), *Lethrinops auritus* (Regan, 1922) (MRAC-A1-049-P-0195-0212_1, MRAC-A1-049-P-0195-0212_2, MRAC-A1-049-P-0195-0212_3, MRAC-A1-049-P-0195-0212_4, MRAC-A1-049-P-0195-0212_5, MRAC-A1-049-P-0195-0212_6, MRAC-A1-049-P-0195-0212_7, MRAC-A1-049-P-0195-0212_8), *Lethrinops marginatus* Ahl, 1926 (ZSM 040856), *Lichnochromis acuticeps* Trewavas, 1935 (ZSM 040891), *Maylandia greshakei* (Meyer and Foerster, 1984) (ZSM 040902), *Maylandia livingstonii* (Boulenger, 1899) (ZSM 040901), *Maylandia lombardoi* (Burgess, 1977) (ZSM 040908), *Maylandia zebra* OB (Boulenger, 1899) (ZSM 041177), *Melanochromis auratus* (Boulenger, 1897) (ZSM 024352_1, ZSM 024352_2), *Melanochromis loriae* Johnson, 1875 (ZSM 041098_5684, ZSM 041098_5683), *Melanochromis* “northern Blue” (ZSM 041174), *Mylochromis lateristriga* (Günther, 1864) (ZSM 041197_5332, ZSM 041197_5333), *Mylochromis melanonotus* (Regan, 1922) (MRAC-81-02-P-28, MRAC-99-041-P-7951), *Mylochromis plagiotaenia* (Regan, 1922) (ZSM 040892), *Naevochromis chrysogaster* (Trewavas, 1935) (ZSM 040860), *Nimbochromis livingstonii* (Günther, 1894) (ZSM 040862), *Nimbochromis polystigma* (Regan, 1922) (ZSM 023156), *Nyassachromis prostoma* (Trewavas, 1935) (ZSM 041204_1, ZSM 041204_2), *Otopharynx lithobates* Oliver, 1989 (ZSM 040903), *Otopharynx* sp. “Nipogo” (ZSM 041180), *Pallidochromis tokolosh* Turner, 1994 (MRAC-99-041-P-10866-10867_1, MRAC-99-041-P-10866-10867_2), *Petrotilapia* indet. (ZSM 041168), *Petrotilapia tridentiger* Trewavas, 1935 (ZSM 024612_1, ZSM 024612_2, ZSM 024612_3), *Placidochromis electra* (Burgess, 1979) (ZSM 040890), *Placidochromis milomo* Oliver, 1989 (ZSM 040872), *Protomelas fenestratus* (Trewavas, 1935) (ZSM 041161_5125, ZSM 041161_5123), *Protomelas taeniolatus* (Trewavas, 1935) (ZSM 040897), *Pseudotropheus elongatus* Fryer, 1956 (ZSM 040896), *Pseudotropheus fuscus* Trewavas, 1935 (ZSM 024696_1, ZSM 024696_2), *Pseudotropheus perspicax* (Trewavas, 1935) (ZSM 024008), *Pseudotropheus socolofi* Johnson, 1974 (ZSM 040009), *Rhamphochromis esox* (Boulenger, 1908) (ZSM 043126), *Rhamphochromis* cf. *esox* (Boulenger, 1908) (ZSM 040874), *Sciaenochromis fryeri* Konings, 1993 (ZSM 040008, ZSM 040899), *Stigmatochromis* “king key” (ZSM 041185), *Stigmatochromis woodi* (Regan, 1922) (MRAC-81-02-P-44-45_1, MRAC-81-02-P-44-45_2, ZSM 042100_5571), *Taeniochromis holotaenia* (Regan, 1922) (ZSM 040904_(P-AA-0563), ZSM 040904), *Taeniolethrinops praeorbitalis* (Regan, 1922) (MRAC-A1-063-P-0012-0013-1, MRAC-A1-063-P-0012-0013-2), *Tramitichromis brevis* (Boulenger, 1908) (ZSM 041193_5156), *Trematocranus placodon* (Regan, 1922) (ZSM 041208_6568), *Tropheops tropheops* (Regan, 1922) (ZSM 041189_5179, ZSM 041189_5176), ZSM 027377_1, ZSM 027377_2, ZSM 027377_3), *Tyrannochromis nigriventer* Eccles, 1989 (ZSM 040898).

Tribe **Haplochromini (*Pseudocrenilabrus*-Group)**: New Kalungwishi Luongo cichlid (Balon and Stewart, 1983) (CU 91753_1, CU 91753_2, CU 91753_3, CU 91753_4, CU 91753_5, CU 91755_1, CU 91755_2, CU 91755_3, CU 91755_4, CU 91755_5, CU 91755_6, CU 91755_7, CU 91755_8), *Pseudocrenilabrus* indet. (ZSM 041496_1, ZSM 041496_2), *Pseudocrenilabrus* new Kalungwishi cichlid (ZSM 041425_1, ZSM 041425_2, ZSM 041425_3, ZSM 041425_4, ZSM 041425_5, ZSM 041425_6, ZSM 041425_7), New Lufubu cichlid (ZSM 041442_1, ZSM 041442_2, ZSM 041442_3, ZSM 041442_4, ZSM 041442_5, ZSM 041442_6, ZSM 041442_7, ZSM 041442_8, ZSM 041442_9), *Pseudocrenilabrus* sp. (ZSM 038206_1, ZSM 038206_2, ZSM 038206_3, ZSM 041473_998, ZSM 041439_1, ZSM 041439_2, ZSM 041439_3, A-0015), *Pseudocrenilabrus multicolor* (Schöller, 1903) (ZSM 041574), *Pseudocrenilabrus nicholsi* (Pellegrin, 1928) (A-0686–1, A-0686–2, A-0686–3), *Pseudocrenilabrus philander* (Weber, 1897) (ZSM 024771, ZSM 040918_577, ZSM 040944_1, ZSM 040944_2, ZSM 040944_3).

Tribe **Haplochromini (Riverine Haplochromini)**: *Astatoreochromis alluaudi* Pellegrin, 1904 (ZSM 041133, ZSM 041146_5932, ZSM 041017–5877), *Ctenochromis pectoralis* Pfeffer, 1893 (ZSM 041480_(DRC-2011+1063), ZSM 041480_(DRC-2011+1062), ZSM 041480_(DRC-2011+1061), ZSM 041461_(DRC-2011+1059), ZSM 041461_(DRC-2011+1058), ZSM 041461_(DRC-2011+1057)), *Haplochromis* sp. aff. *bakongo* (ZSM 037741_1, ZSM 037741_2), *Haplochromis bloyeti* (Sauvage, 1883) (MRAC -97-068-P-0021-0022_1, MRAC -97-068-P-0021-0022_2, MRAC -97-068-P-0021-0022_3, MRAC -78-19-P-236-239_1, MRAC -78-19-P-236-239_2, MRAC -78-19-P-236-239_3, MRAC -78-19-P-236-239_4, MRAC -119803-808_1, MRAC -119803-808_2, MRAC -119803-808_3, MRAC -119803-808_4), *Haplochromis burtoni* (Günther, 1894) (ZSM 040835, IRSNB A-0011_1, IRSNB A-0011_2), *Haplochromis cf. demeusii* (ZSM 037760), *Haplochromis demeusii* (Boulenger, 1899) (ZSM 035299_(US-CAM-2005+1), ZSM 035299_(US-CAM-2005+2), ZSM 038137_1, ZSM 038137_2, ZSM 038137_3, ZSM 038137_4, ZSM 038137_5, ZSM 038137_6, ZSM 038137_7, ZSM 038137_8, ZSM 038137_9, ZSM 038137_(DRC-2008+257)), *Haplochromis desfontainii* (Lacépède, 1802) (ZSM 023690_1, ZSM 023690_2, ZSM 023690_3), *Haplochromis flaviijosephi* (Lortet, 1883) (ZSM 040028_1, ZSM 040028_2, ZSM 040028_3, ZSM 040028_4, ZSM 040028_5, ZSM 040030_1, ZSM 040030_2, ZSM 040030_3, ZSM 040030_4), *Haplochromis katonga* Schraml and Tichy, 2010 (ZSM 030363_(1998+8680), ZSM 030363_(1998+8678)), *Schwetzochromis neodon* Poll, 1948 (RG 79591-79644_1, RG 79591-79644_2, RG 79591-79644_3, RG 79591-79644_4, RG 79591-79644_5, RG 79591-79644_6, RG 79591-79644_7, RG 79591-79644_8, ZSM 96-031-P-1303-1307_1, ZSM 96-031-P-1303-1307_2, ZSM 96-031-P-1303-1307_3, ZSM 96-031-P-1303-1307_4, ZSM 96-031-P-1303-1307_5, ZSM 96-031-P-1308-1309_2).

Tribe **Haplochromini (Serranochromines):** *Haplochromis polli* Thys van den Audenaerde, 1964 (MRAC_98258, ZSM 024002_1, ZSM 024002_2, ZSM 024002_3, ZSM 035600_4, ZSM 035600_5, ZSM 038133_(DRC-2008+422), ZSM 038133_(DRC-2008+423)), *Haplochromis fasciatus* (Perugia, 1892) (ZSM 035297_1), *Sargochromis* sp. (ZSM 042232_(DRC-2012+1515), ZSM 042232_(DRC-2012+1514)), *Serranochromis* cf. *thumbergi* (Castelnau, 1861) (ZSM 042230, ZSM 041426_3, ZSM 041426_(DRC-2011+1006), ZSM 041426_(DRC-2011+1005)).

Tribe **Haplochromini (Lake Victoria Haplochromini):** *Haplochromis ampullarostratus* Schraml, 2004 (ZSM 029759_(1999+9620), ZSM 029759_(1999+ES99_1)), *Haplochromis chilotes* (Boulenger, 1911) (ZSM 040867), *Haplochromis commutabilis* Schraml, 2004 (ZSM 029761_(1999+9645), ZSM 029761_(1999+9667)), *Haplochromis exspectatus* Schraml, 2004 (ZSM 029757_(1999+9602), ZSM 029757_(1999+9679)), *Haplochromis ishmaeli* Boulenger, 1906 (ZSM 040864), *Haplochromis luteus* (Seehausen and Bouton, 1998) (ZSM 040888), *Haplochromis nyererei* Witte-Maas and Witte, 1985 (ZSM 040863), *Haplochromis obtusidens* Trewavas, 1928 (ZSM 041139), *Haplochromis omnicaeruleus* (Seehausen and Bouton, 1998) (ZSM 040868), *Haplochromis plagiodon* Regan and Trewavas, 1928 (ZSM 041134_1, ZSM 041134_2, ZSM 041134_3), *Haplochromis retrodens* (Hilgendorf, 1888) (ZSM 023474_1, ZSM 023474_2), *Haplochromis rudolfianus* Trewavas, 1933 (ZSM 025097_1, ZSM 025097_2), *Haplochromis squamipinnis* Regan, 1921 (ZSM 030692, ZSM 030693).
