# Supplementary figures and images for: New haplochromine cichlid from the upper Miocene (9–10 MYA) of Central Kenya
Source: BMC Evol Biol. 2020 Jun 5;20:65. doi: 10.1186/s12862-020-01602-x (PMC7275555; doi:10.1186/s12862-020-01602-x)

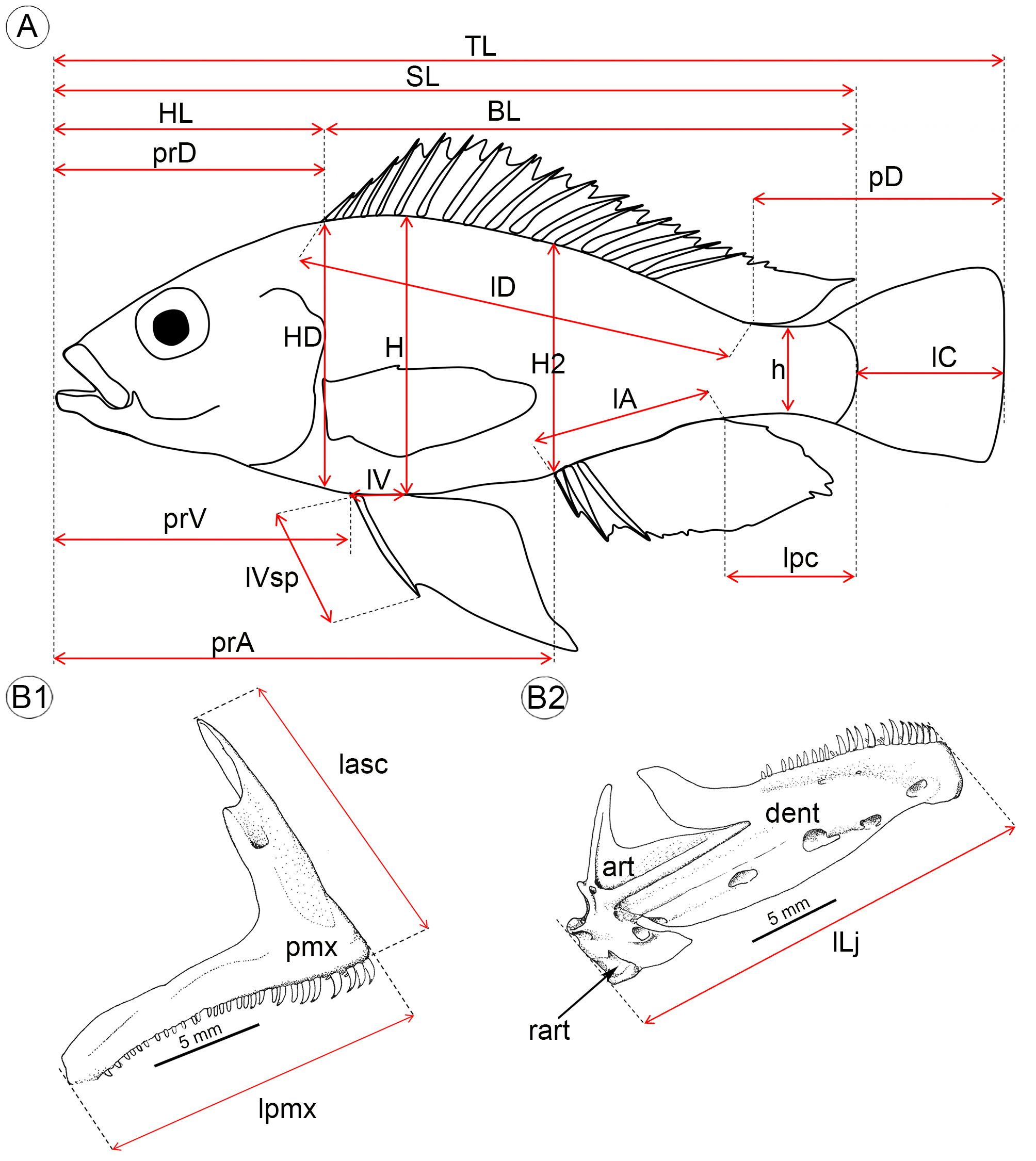

Supplement: Supplementary file 3 — Additional file 3. Supplementary Fig. S1. Morphometric measurements conducted for this study. A, generalized cichlid fish depicting head-, body-, and fin-related linear measurements (re-drawn based on de Zeeuw et al., 2010 [114], Fig. 14 distributed under CC-BY license (https://creativecommons.org/licenses/by/4.0/) with permission from Naturalis Biodiversity Center https://www.repository.naturalis.nl/record/358750). B1–2, Upper and lower jaw bones (right side, lateral view) of a haplochromine cichlid with measurements (modified from Van Oijen & de Zeeuw, 2008] [194]: Figs. 3 & 6., based on Haplochromis vonlinnei, distributed under CC-BY license (https://creativecommons.org/licenses/by/4.0/) with permission from Naturalis Biodiversity Center http://www.repository.naturalis.nl/record/261776). Abbreviations: art, angulo-articular; BL, body length; h, minimal body height; dent, dentary; H, maximal body height; H2, body height at origin of anal fin; HD, head depth; HL, Head length; lA, length of anal fin base; lasc, length of premaxillary ascending process; lC, length of caudal fin; lD, length of dorsal fin base; lLj, length of lower jaw; lpc, length of caudal peduncle; lpmx, length of premaxilla; lV, length of pelvic fin base; lVsp, length of pelvic fin spine; pD, postdorsal distance; pmx, premaxilla; prA, preanal distance; prD, predorsal distance; prV, prepelvic distance; rart, retro-articular; SL, standard length; TL, total length. [file 12862_2020_1602_MOESM3_ESM.tif]

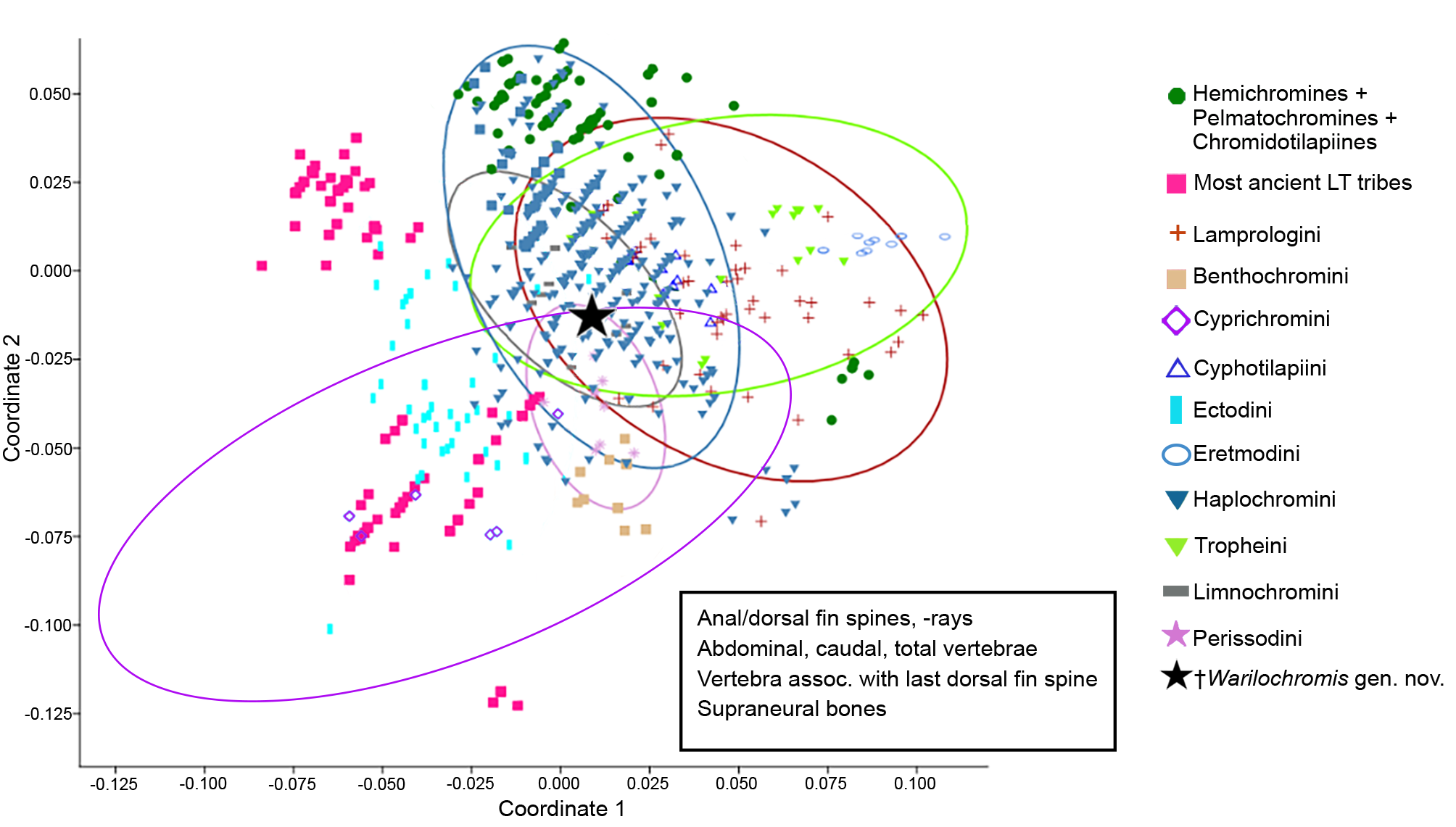

Supplement: Supplementary file 6 — Additional file 6 Supplementary Fig. S2. Principal coordinates analysis (PCoA) scatter plot. Analysis based on eight meristic characters and the number of supraneurals from the 16 pseudocrenilabrine tribes (26 lineages) shown in Fig. 7 (N = 854; see Additional file 2: Supplementary Data S2 for raw data) and from †Warilochromis unicuspidatus gen. et sp. nov. Species score limits are visualized as 95% confidence ellipses. Note that the ellipses shown encompass only members of the tribes that overlap with †Warilochromis. Coordinate 1 explains 47.13% and Coordinate 2 explains 40.75% of the variation. [file 12862_2020_1602_MOESM6_ESM.tif]
