# Supplementary material for: New haplochromine cichlid from the upper Miocene (9–10 MYA) of Central Kenya
Source: BMC Evol Biol. 2020 Jun 5;20:65. doi: 10.1186/s12862-020-01602-x (PMC7275555; doi:10.1186/s12862-020-01602-x)
Supplement: Supplementary file 5 — Additional file 5. Character list and states used for phylogenetic analysis (compiled from Stiassny, [85]). [file 12862_2020_1602_MOESM5_ESM.doc]

**Additional file 4: Character list and states used for phylogenetic analysis (compiled from Stiassny [1])**

**Character 1:** **Length of arms of first epibranchial (Ep1):** *(0) uncinate process shorter than anterior arm; (1) subequal or uncinate process longer than anterior arm*.

**Character 2:** **2nd epibranchial toothplates:** *(0) present; (1) absent*.

**Character 3:** **4th ceratobranchial toothplates:** *(0) attached to gill rakers; (1) separate from gill rakers*.

**Character 4:** **NLF0 foramina:** *(0) NLF0 widely separated from one another; (1) borne at the end of short, medially directed tubes and partially (or fully) coalesced in the midline*.

**Character 5:** **Overlap between NLF0 foramina and supraoccipital crest (=supraoccipital in Stiassny [1]):** *(0) NLF0 not overlain by supraoccipital crest; (1) NLF0 overlain by supraoccipital crest*.

**Character 6:** **Central ligament:** *(0) bifurcate and passes from rostral tip of the LPJ to the third hypobranchial elements; (1) single band that passes from the LPJ to the fourth basibranchial element*.

**Character 7: Frontal canals:** *(0) absent; (1) paired frontal canals united in the midline and extending rostrodorsally as single central tube*.

**Character 8:** **Suture between vomerine wing and parasphenoid:** *(0) simple sutural union; (1) strongly interdigitated suture*.

**Character 9: Origin of pharyngocleithralis muscle on cleithrum:** *(0) originates from the medial aspect of the cleithrum; (1) originates from lateral face of cleithrum*.

**Character 10: Urohyal:** *(0) with no projection on anterodorsal surface (= with no rostrally directed spine in Stiassny, [1]); (1) with projection on anterodorsal surface*.

**Character 11:** **Number of vertebrae:** *(0) not increased; (1) increased*.

**Character 12: Obliquus inferioris muscle:** *(0) no subdivision of the muscle; (1) associated with first and second postcleithra*.

**Character 13:** **Vomer:** *(0) not notched; (1) characteristically notched*.

**Character 14:** **Sphenotic foramina:** *(0) not enlarged or absent; (1) enlarged*.

**Character 15:** **Microbranchiospines:** *(0) small, spines restricted to lateral margins; (1) large, spines on the exposed surface*.

**Character 16:** **Pharyngocleithralis internus muscle:** *(0) inserts musculously or via broad bands on 5th ceratobranchial (cb); (1) inserts onto LPJ via single cord-like tendon*.

**Character 17:** **Lateral line foramina on preopercle** (=Preopercular pores in Stiassny, [1])**:** *(0) 7; (1) 6*.

**Character 18:** **Lateral line foramina on dentary** (=Dentary pores in Stiassny, [1]**):** *(0) 5; (1) 4*.

**Character 19: Predorsal scale pattern:** *(0) stochastic; (1) uni- or tri-serial*.

**Character 20:** **Egg laying pattern:** *(0) loose, or open egg plaques; (1) tight circular plaque*.

**Character 21:** **Adductor arcus palatini muscle:** *(0) limited to pterygoid series because fossa is lacking; (1) extends to insert onto palatine in region of palatine fossa*.

**Character 22: Palato-maxillary ligament:** *(0) palatine prong attached to maxillary saddle by short ligament; (1) well-developed and strap-like, extends much of length of palatine prong*.

**Character 23:** **Pharyngocleithralis internus muscle insertion onto LPJ:** *(0) broad musculous association; (1) via single cord-like tendon*.

**Character 24: Uncinate process of Ep1:** *(0) two arms of Ep1 subequal in length (uncinate process shorter than anterior arm); (1) directed caudad and longer than anterior arm*.

**Character 25: Supraneurals** (=predorsal bones in Stiassny, [1])**:** *(0) 2; (1) 1*.

**Character 26: Opercular spot:** *(0) absent; (1) present*.

**Character 27:** **Lacrimal** (=lachrymal in Stiassny, [1])**:** *(0) two lacrimals; (1) single lacrimal followed by tubular 2nd infraorbital (io2)*.

**Character 28: Foramina in exoccipital bones**: *(0) small; (1) large*.

**Reference**

1 Stiassny MLJ. Phylogenetic intrarelationships of the family Cichlidae. In: Cichlid fishes*.* Edited by Keenleyside MHA. London: Chapman & Hall; 1991: 1–35.
